# Plant-derived extracellular vesicles deliver spermidine to reprogram macrophage metabolism and ameliorate rheumatoid arthritis

**DOI:** 10.1186/s12951-026-04836-x

**Published:** 2026-07-26

**Authors:** Xiaocheng Wang, Jiaxin He, Yixiang Hong, Xingyue Zeng, Min Li, Shixiong Cao, Renge Liang, Gengmin Zhou, Qingwen Wang

**Affiliations:** 1 Department of Rheumatism and Immunology, Peking University Shenzhen Hospital, Shenzhen, 518036 China; 2 Institute of Immunology and Inflammatory Diseases, Shenzhen Peking University-The Hong Kong University of Science and Technology Medical Center, Shenzhen, 518036 China; 3 Shenzhen Key Laboratory of Inflammatory and Immunology Diseases, Shenzhen, 518036 China; 4 Department of Rheumatology & Immunology, Beijing Key Laboratory for Rheumatism Mechanism and Immune Diagnosis (BZ0135), Peking University People’s Hospital, Beijing, 100044 China

**Keywords:** Rheumatoid arthritis, Red cabbage-derived vesicles, Macrophage metabolic reprogramming, Spermidine, PI3K-AKT signaling

## Abstract

Rheumatoid arthritis (RA) is driven by macrophage-mediated synovial inflammation closely linked to metabolic dysfunction, highlighting immunometabolism as a therapeutic target. Here, we engineered spermidine-loaded red cabbage-derived extracellular vesicles (RCEVs@SPD) as a plant-based nanoplatform to reprogram macrophage bioenergetics. In LPS-stimulated macrophages, RCEVs@SPD suppressed pro-inflammatory polarization and enhanced mitochondrial function, as evidenced by increased ATP production and mitochondrial membrane potential. Assay-based measurements further showed reduced extracellular acidification rate (ECAR) and elevated oxygen consumption rate (OCR), indicating a shift from glycolysis toward oxidative phosphorylation (OXPHOS). Untargeted LC-MS metabolomics revealed coordinated alterations in pathways supporting mitochondrial cofactor biosynthesis, redox balance, and central carbon metabolism. Transcriptomic analysis confirmed enrichment of OXPHOS and mitochondrial respiration gene signatures with concomitant repression of glycolytic programs and PI3K-AKT signaling. In vivo, RCEVs@SPD significantly alleviated collagen-induced arthritis, improved joint histopathology, and promoted anti‑inflammatory macrophage polarization. Notably, systemic macrophage depletion abolished the additional therapeutic benefit of RCEVs@SPD, demonstrating macrophage dependence. Collectively, these findings establish plant-derived extracellular vesicles as a biocompatible delivery system that potentiates spermidine to restore macrophage metabolic balance and mitigate RA pathology.

**Graphical Abstract:**

**Figure:**
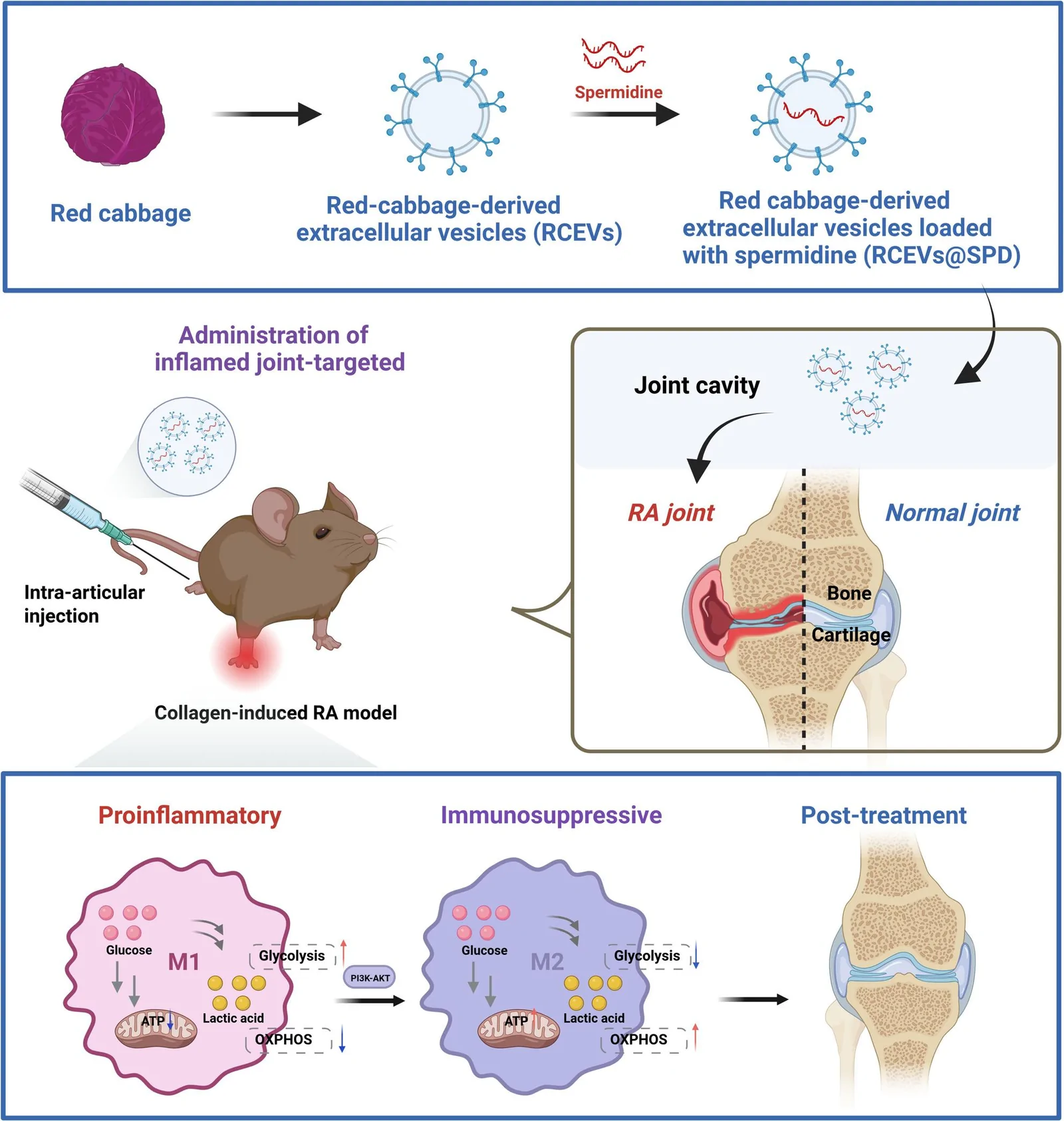

**Supplementary Information:**

The online version contains supplementary material available at https://doi.org/10.1186/s12951-026-04836-x.

## Introduction

Rheumatoid arthritis (RA) is a chronic autoimmune disease characterized by persistent synovial inflammation, progressive cartilage degradation, and irreversible bone erosion [1–3]. Despite major advances in disease-modifying antirheumatic drugs (DMARDs) and biologic therapies, a substantial proportion of patients fail to achieve durable remission or prone to systemic adverse effects associated with long-term immunosuppression [4, 5]. These limitations underscore the need for therapeutic strategies that extend beyond broad inflammatory blockade to restore the dysregulated cellular programs that sustain disease progression.

A growing body of evidence identifies metabolic reprogramming as a fundamental regulator of immune and stromal cell function in RA. Within the inflamed synovium, proinflammatory macrophages and pathogenic fibroblast-like synoviocytes (FLS) preferentially adopt glycolysis-dominant metabolism even under normoxic conditions, a phenotype reminiscent of the Warburg effect [6–8]. This metabolic configuration supports inflammatory cytokine production, extracellular matrix remodeling, and immune cell recruitment, thereby reinforcing chronic synovitis [9, 10]. In contrast, anti-inflammatory and tissue-repair phenotypes are closely linked to intact mitochondrial respiration and oxidative phosphorylation (OXPHOS), positioning cellular metabolism as an upstream and actionable driver of synovial pathology [11, 12].

Recent studies demonstrate that targeted manipulation of cellular metabolism can effectively modulate synovial inflammation and disease severity [13, 14]. External physical or chemical cues capable of rewiring metabolic pathways have shown promise in suppressing pathogenic activation of synoviocytes and immune cells, supporting a paradigm in which metabolic control is not merely a downstream consequence of inflammation but a determinant of inflammatory fate [15]. However, many reported approaches rely on invasive procedures, specialized equipment, or non-physiological stimulation, which limits their translational potential.

Naturally occurring metabolites have therefore attracted increasing interest as immunometabolic modulators due to their intrinsic biocompatibility and pleiotropic biological functions. Spermidine (SPD), a ubiquitous dietary polyamine, has been implicated in autophagy induction, mitochondrial quality control, and inflammation resolution across diverse disease contexts [16–18]. Nevertheless, the therapeutic application of free SPD is constrained by rapid systemic clearance, limited tissue retention, and lack of targeted delivery to inflamed sites, highlighting the need for an effective delivery platform.

Plant-derived extracellular vesicles (PDEVs) have recently emerged as a bioinspired class of nanocarriers with favorable safety profiles, intrinsic stability, and inherent biological activity [19, 20]. Unlike synthetic nanoparticles, PDEVs originate from edible plants and can transfer functional biomolecules to mammalian cells, enabling cross-kingdom communication with minimal biosafety concerns [21–23]. Among them, red cabbage-derived extracellular vesicles (RCEVs) are particularly attractive due to their scalable availability and metabolically relevant cargo. We hypothesized that encapsulating SPD within RCEVs could provide a synergistic strategy for localized, metabolism-oriented immunomodulation in RA.

Macrophage metabolic state is tightly coupled to intracellular signaling networks, including PI3K-AKT-dependent reinforcement of glycolysis and energy-sensing pathways such as AMPK and FoxO that favor mitochondrial metabolism [24, 25]. We postulated that RCEV-mediated delivery of SPD could alleviate glycolytic pressure and redirect metabolic flux toward OXPHOS through coordinated modulation of these pathways, thereby restoring macrophage metabolic homeostasis.

In this study, we engineered spermidine-loaded red cabbage-derived extracellular vesicles (RCEVs@SPD) and systematically evaluated their immunometabolic and therapeutic effects in RA. Using integrated functional assays, transcriptomic profiling, and untargeted LC-MS metabolomics, we demonstrate that RCEVs@SPD reprogram macrophage metabolism away from glycolysis and toward mitochondrial respiration, accompanied by broad remodeling of inflammatory and metabolic networks. Importantly, this macrophage-directed metabolic intervention translated into robust therapeutic benefit in the collagen-induced arthritis model, with attenuation of joint inflammation and structural damage and a favorable systemic safety profile. Together, these findings establish a plant-derived vesicle-based metabolic reprogramming strategy that complements existing RA therapies by restoring immune metabolic balance rather than enforcing broad immunosuppression.

## Materials and methods

For clarity and to meet manuscript length requirements, comprehensive experimental protocols are presented in the Supplementary Materials. These include full reagent information (catalog numbers and working concentrations), detailed cell and animal handling procedures, instrument specifications and settings, primer sequences, antibody panels with dilutions, imaging acquisition and processing pipelines, as well as data preprocessing steps, statistical analyses, and replication/quality-control standards.

## Results

### Metabolic reprogramming toward glycolysis is a defining feature of synovial macrophages in RA

**Fig. 1:**
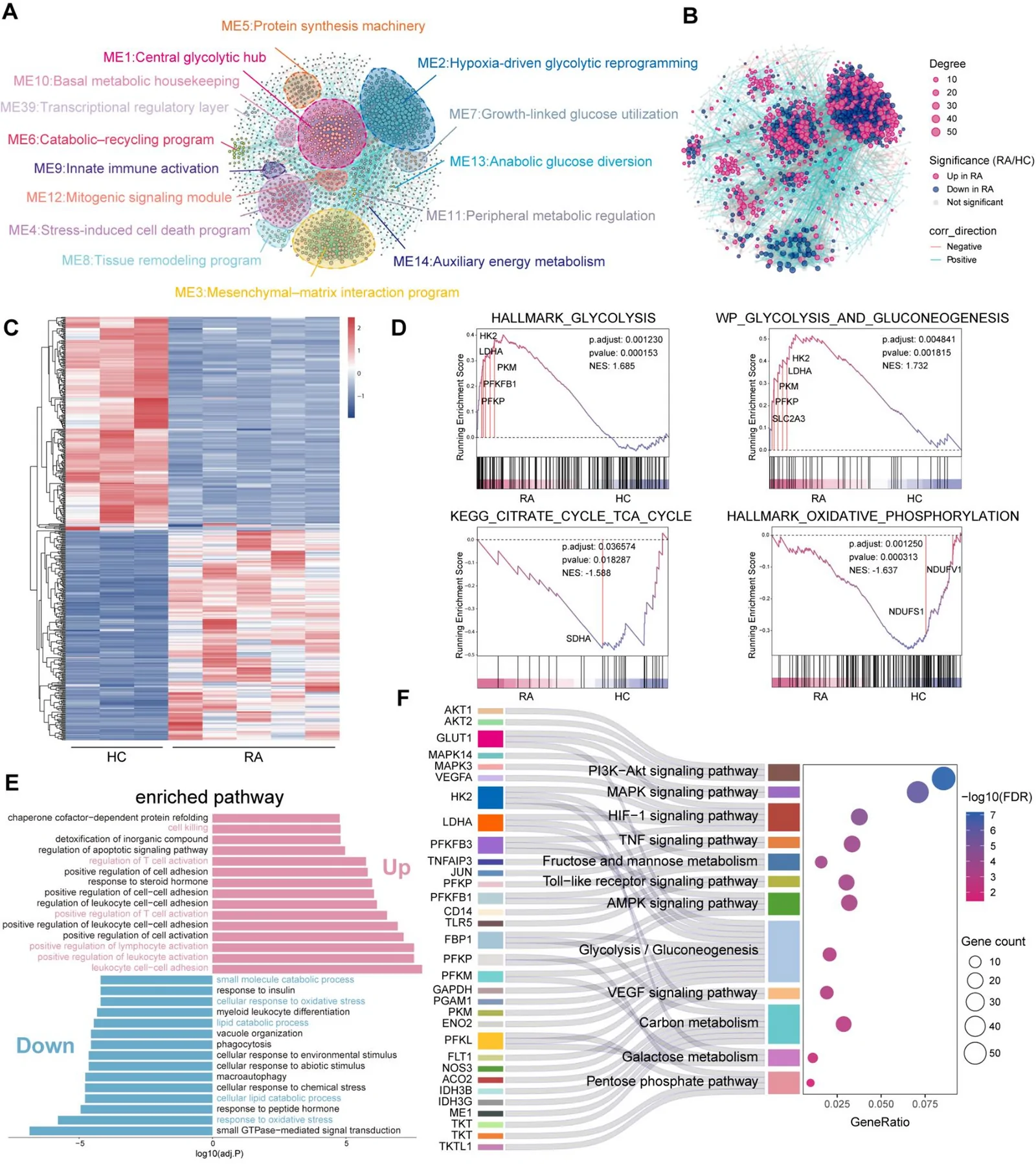
Gene expression characteristics and functional enrichment analysis in RA and HC. (**A**) Functional modules identified by WGCNA. (**B**) DEGs significantly upregulated or downregulated in RA compared with HC overlaid onto the co‑expression network to illustrate their module distribution. (**C**) Heatmap of DEGs identified by RNA‑seq, showing expression patterns across samples and group separation. (**D**) Gene set enrichment analysis (GSEA) of DEGs, highlighting significantly enriched pathways and gene sets. (**E**) GO enrichment analysis of DEGs, summarizing enriched biological processes (BP), molecular functions (MF), and cellular components (CC). (**F**) KEGG pathway network derived from enrichment analysis, where each node represents a KEGG pathway, node size corresponds to the number of enriched genes in the pathway, and edge thickness reflects the number of shared genes between pathways

To characterize the metabolic state of synovial macrophages in RA, we performed WGCNA on RNA-seq data from the GSE10500 dataset (Fig. 1A). Fifteen co-expression modules were identified, several of which were significantly upregulated in RA macrophages, including modules associated with central glycolysis, hypoxia-driven glycolytic reprogramming, protein synthesis, catabolic recycling, innate immune activation, and mitotic signaling (Fig. 1B). Functional annotation revealed a coordinated enhancement of metabolic and inflammatory programs.

Notably, glycolysis-related modules were enriched for core glycolytic enzymes and hypoxia-responsive pathways, indicating increased glycolytic flux and metabolic adaptation to the hypoxic synovial microenvironment. In parallel, modules linked to innate immune activation and cell proliferation were upregulated, highlighting a tight coupling between inflammatory signaling and metabolic demand. Enhanced activity of translation- and catabolism-related modules further suggests that glycolysis supports sustained inflammatory protein production and substrate supply under chronic inflammatory stress.

Differential expression analysis between RA and healthy control (HC) macrophages confirmed these findings (Fig. 1C). Gene set enrichment analysis (GSEA) demonstrated significant enrichment of glycolysis-related pathways, including HALLMARK GLYCOLYSIS and WP_GLYCOLYSIS_AND_GLUCONEOGENESIS, in RA macrophages (Fig. 1D), driven by key genes such as HK2, LDHA, PFKFB1, PFKP, PKM, and SLC2A3. In contrast, mitochondrial energy metabolism pathways, including the tricarboxylic acid cycle and oxidative phosphorylation, were significantly downregulated, with enrichment biased toward HC samples. GO analysis further showed that upregulated genes in RA macrophages were primarily associated with leukocyte activation and cell adhesion, processes known to require elevated glycolytic metabolism. Conversely, downregulated genes were enriched in oxidative and catabolic processes, indicating suppression of mitochondrial oxidative metabolism (Fig. 1E).

Integration of differentially expressed genes and KEGG pathways using Sankey analysis revealed a highly centralized metabolic network focused on glycolysis. Most RA-associated genes converged on the glycolysis/gluconeogenesis pathway and extended to related pathways such as the pentose phosphate pathway and carbon metabolism. Key nodes included SLC2A1, HK2, PFKFB1, PFKFB3, PFKP, PKM, GAPDH, and LDHA, spanning glucose uptake through pyruvate generation. Collectively, these results demonstrate that synovial macrophages in RA undergo a coordinated metabolic reprogramming toward glycolysis, defining a macrophage activation state adapted to the hypoxic and inflammatory joint microenvironment (Fig. 1F).

To verify that inflammatory activation contributes to this metabolic profile, RAW264.7 macrophages were polarized to an M1 phenotype and analyzed by qPCR. M1 macrophages showed strong suppression of OXPHOS-related genes including NDUFA9, NDUFB8, COX4I1, COX5B, ATP5A, ATP5B, PGC-1α and TFAM, together with increased expression of glycolytic genes such as GLUT1, PFKFB3, HK2, PKM2 and LDHA, as well as the inflammatory marker iNOS (Figure S1). These changes closely paralleled the transcriptional patterns observed in RA synovial macrophages.

In vivo validation using CIA mice further confirmed this metabolic shift. CIA joints displayed markedly increased iNOS and elevated GLUT1 expression (Figure S2A, B), supporting enhanced glycolysis within the inflamed synovial microenvironment. Collectively, these data identify enhanced glycolysis and suppressed OXPHOS as defining metabolic features of inflammatory macrophages in RA.

### Isolation, characterization, and biocompatibility of RCEVs

To compare the effects of different isolation strategies on the quality of RCEVs, two extraction workflows were established, including conventional ultracentrifugation and sucrose cushion ultracentrifugation (Table S3). Both methods yielded nanoscale vesicles with typical morphology, whereas the sucrose cushion method demonstrated improved purity. Endotoxin analysis revealed that the final RCEV preparations contained 0.173 ± 0.014 EU/mL (mean ± SD, *n* = 6), indicating minimal endotoxin contamination and ensuring suitability for subsequent immunological experiments.

**Fig. 2:**
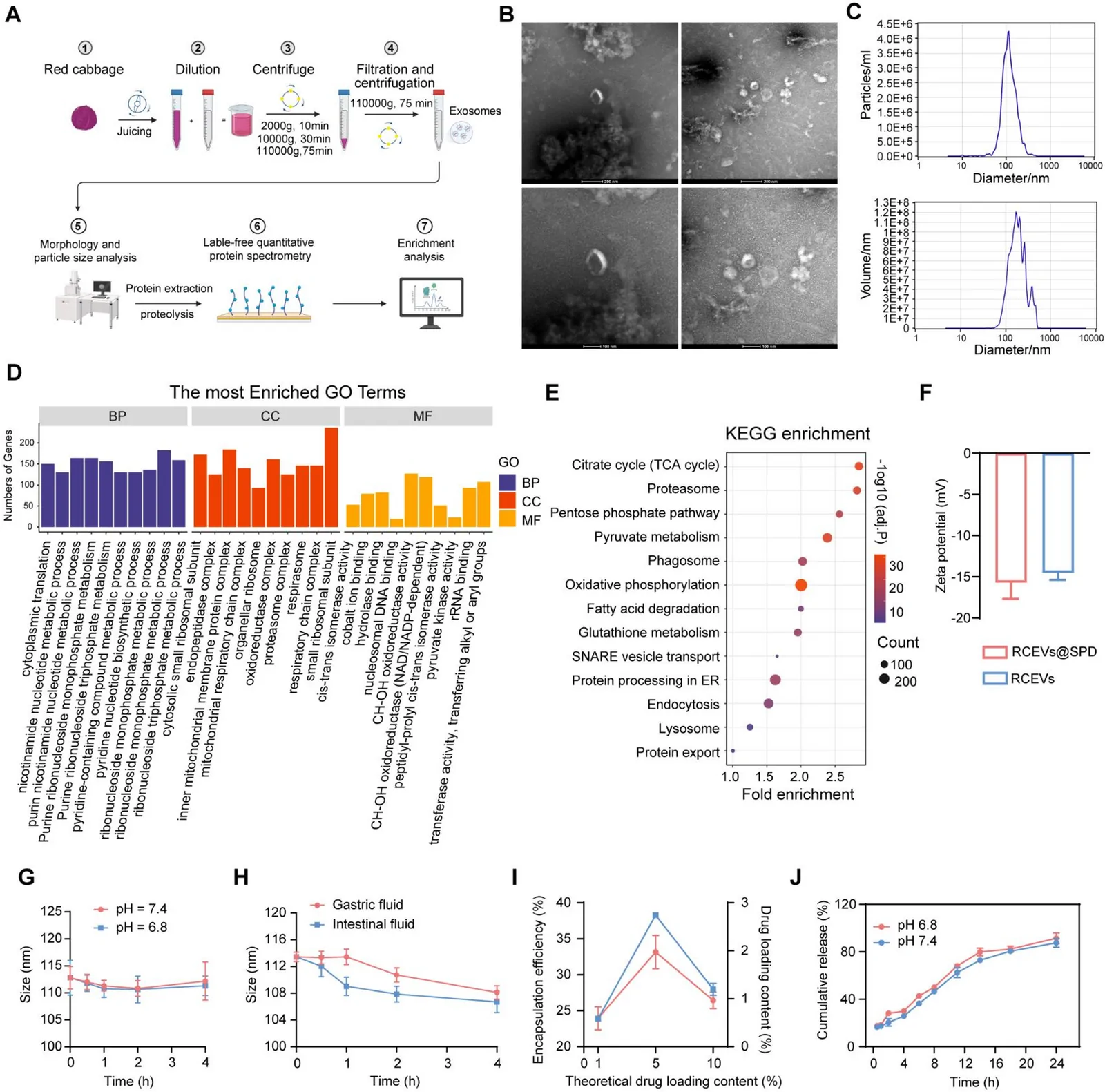
Preparation, characterization, and functional analysis of RCEVs. (**A**) Schematic illustration of the isolation procedure of RCEVs from red cabbage and the subsequent label-free quantitative proteomics workflow. (**B**) TEM images showing the typical vesicular morphology of RCEVs. (**C**) Particle size distribution of RCEVs measured by NTA. (**D, E**) GO and KEGG enrichment analyses based on label-free proteomic profiling of RCEVs. (**F**) Zeta potential measurements of RCEVs and RCEVs@SPD. (**G**) Size stability of RCEVs@SPD under different pH conditions. (**H**) Size stability of RCEVs in simulated gastrointestinal fluids. (**I**) Encapsulation efficiency and drug loading content of RCEVs@SPD at different theoretical loading ratios. (**J**) In vitro cumulative release profiles of RCEVs@SPD under different pH conditions. All quantitative data are presented as mean ± SD. Statistical analyses were performed using one‑way ANOVA followed by Tukey’s multiple comparison test. Differences were considered statistically significant at *p < 0.05

Purity was further assessed by comparing nanoparticle tracking analysis (NTA) particle size distribution and concentration before and after membrane lysis. For RCEVs isolated by ultracentrifugation (Figure S3A), particle concentration markedly decreased after lysis, confirming that the majority of detected particles were membrane-enclosed vesicles. Similarly, RCEVs isolated via sucrose cushion ultracentrifugation (Figure S3B) showed a pronounced reduction in particle counts following lysis, with a lower proportion of residual particles, suggesting reduced contamination by non-membranous particles and improved purity. Based on these comparative results, the optimized isolation method was employed for subsequent experiments (Fig. 2A).

Transmission electron microscopy (TEM) revealed typical cup-shaped or spherical vesicular morphology (Fig. 2B). NTA demonstrated a relatively homogeneous particle size distribution within the nanoscale range (Fig. 2C). To characterize their molecular composition, label-free quantitative proteomics was performed. GO enrichment analysis indicated that RCEV-associated proteins were predominantly enriched in vesicle-mediated transport and metabolic regulation (Fig. 2D). KEGG pathway analysis further identified significant enrichment in ribosome, glycolysis/gluconeogenesis, oxidative phosphorylation, and cellular stress-related pathways (Fig. 2E), suggesting that RCEVs carry metabolically active bioactive cargos.

Physicochemical characterization showed that both native RCEVs and RCEVs@SPD exhibited negatively charged surface potentials. A modest shift in zeta potential after drug loading indicated successful encapsulation without compromising vesicle integrity (Fig. 2F). Moreover, RCEVs@SPD maintained stable particle size distributions under different pH conditions (Fig. 2G) and in simulated gastric and intestinal fluids (Fig. 2H), supporting their potential suitability for oral delivery.

Drug loading efficiency was evaluated using different theoretical SPD-to-RCEV input ratios (1:1, 1:5, and 1:10). The encapsulation efficiency (EE%) and drug loading content (DLC%) were calculated based on SPD quantification using the UV-Vis standard calibration curve shown in Figure S4. Among the tested ratios, the 1:5 formulation achieved an optimal balance between encapsulation efficiency and loading capacity (Fig. 2I). At this optimized ratio, the encapsulation efficiency and drug loading content were 33.2 ± 2.3% and 2.79 ± 0.05%, respectively (mean ± SD, *n* = 3). This ratio was therefore selected for subsequent experiments. In vitro release assays demonstrated sustained SPD release from RCEVs@SPD under different pH conditions (Fig. 2J). Biocompatibility evaluation using a CCK‑8 assay showed that RCEVs did not induce significant cytotoxicity in RAW264.7 macrophages across the tested concentrations and time points (Figure S5).

To further determine whether RCEVs enhance intracellular delivery of SPD, the intracellular SPD concentration in RAW264.7 macrophages was quantified following treatment with free SPD or RCEVs@SPD at equivalent SPD doses. Compared with free SPD, RCEVs@SPD treatment resulted in significantly higher intracellular SPD accumulation, indicating improved cellular delivery efficiency mediated by vesicular encapsulation (Figure S6). These findings suggest that RCEVs not only provide sustained release but also facilitate enhanced intracellular transport of SPD, thereby supporting their function as effective nano-carriers.

### In vitro cellular uptake and in vivo distribution of RCEVs

**Fig. 3:**
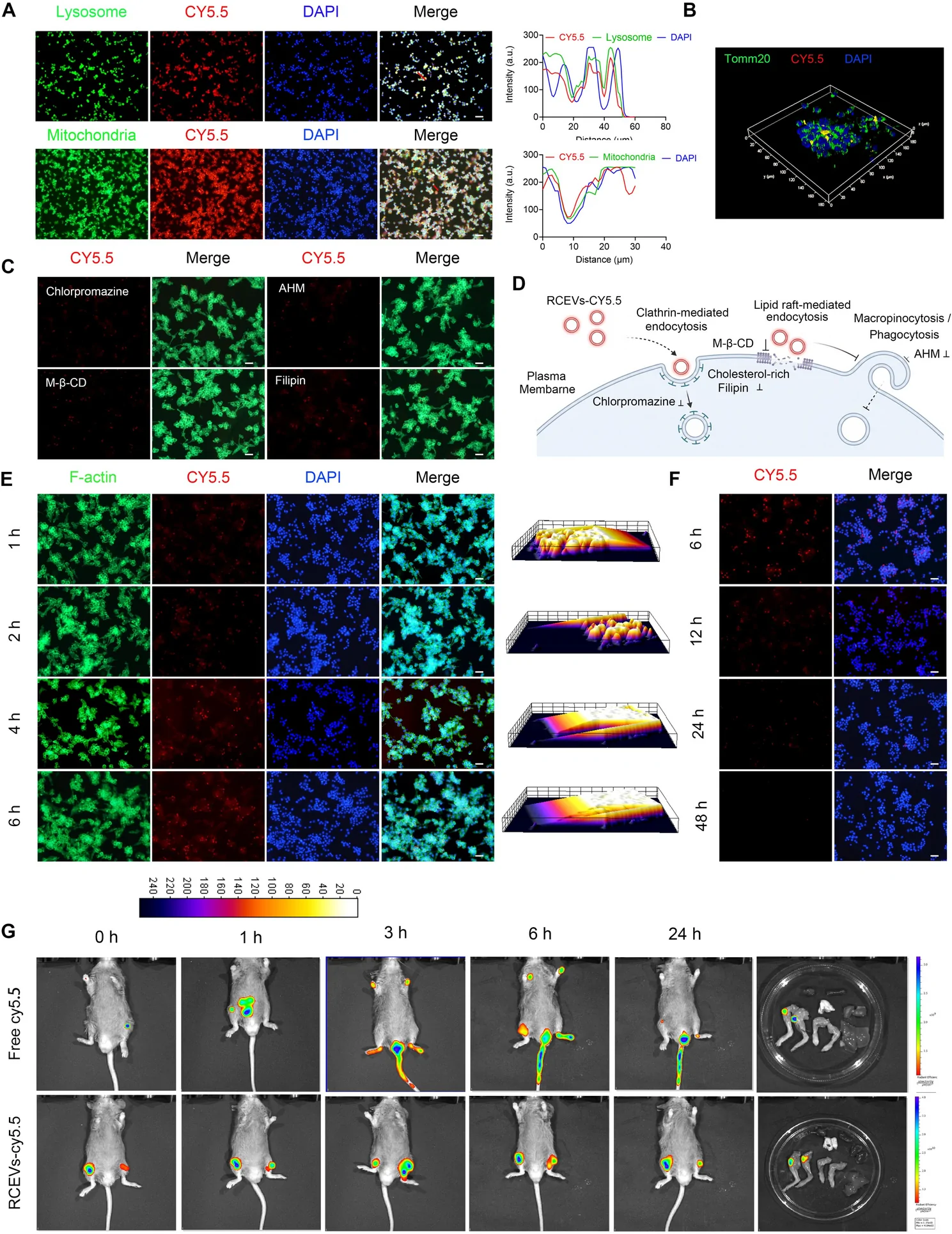
Cellular uptake, intracellular trafficking, and in vivo biodistribution of CY5.5-labeled RCEVs. (**A**) Fluorescence microscopy images showing the intracellular localization of CY5.5-labeled RCEVs (red) in RAW264.7 co-stained with lysosome tracker (green, upper panel) or mitochondria tracker (green, lower panel). Nuclei were counterstained with DAPI (blue). (**B**) Three-dimensional reconstruction of intracellular CY5.5-labeled RCEVs distribution co-stained with Tom20-labeled mitochondria, highlighting spatial localization patterns. (**C**) Effects of different endocytosis inhibitors on the cellular uptake of CY5.5-labeled RCEVs, including chlorpromazine, Mβ-CD, filipin, and AHM. (**D**) Schematic illustration of the proposed internalization pathways of RCEVs, including clathrin-mediated endocytosis, lipid raft-mediated endocytosis, and macropinocytosis. (**E**) Time-dependent intracellular distribution of CY5.5-labeled RCEVs at 1, 2, 4, and 6 h. F-actin (green) was used to visualize cytoskeletal structures. Three-dimensional fluorescence intensity surface plots (right) depict intracellular signal accumulation over time. (**F**) Long-term intracellular retention of CY5.5-labeled RCEVs at 6, 12, 24, and 48 h post-incubation, demonstrating sustained intracellular presence. (**G**) In vivo fluorescence imaging of mice following administration of free CY5.5 or RCEVs‑CY5.5 at 0, 1, 3, 6, and 24 h. Ex vivo fluorescence imaging of major organs is shown on the right, revealing differential biodistribution and retention profiles mediated by RCEVs. Data are presented as mean ± SEM

To investigate intracellular trafficking of RCEVs, CY5.5-labeled RCEVs were incubated with RAW264.7 macrophages and analyzed by confocal microscopy. RCEVs exhibited minimal colocalization with lysosomes but showed pronounced spatial association with mitochondria (Fig. 3A). Line‑scan profiling and three‑dimensional reconstruction consistently confirmed limited lysosomal sequestration and preferential mitochondrial proximity (Fig. 3B), suggesting potential mitochondrial involvement following internalization.

Pharmacological inhibition studies demonstrated that RCEV uptake was significantly reduced by chlorpromazine, methyl‑β‑cyclodextrin (M‑β‑CD), filipin, and amiloride hydrochloride (AHM), indicating the involvement of clathrin‑mediated endocytosis, lipid raft-dependent pathways, and macropinocytosis/phagocytosis in the internalization process (Fig. 3C, D). Importantly, cell viability assays excluded cytotoxic effects of these inhibitors (Figure S7), confirming that the reduced fluorescence intensity resulted from impaired endocytic activity rather than decreased cell viability. Time-dependent imaging revealed progressive intracellular accumulation of RCEVs within 1–6 h, followed by sustained intracellular retention up to 48 h (Fig. 3E, F), suggesting dynamic uptake accompanied by prolonged intracellular persistence.

Consistent with these findings, efficient uptake of CY5.5‑labeled RCEVs was also observed in synovial fibroblast‑like cells (Figure S8), indicating that RCEV internalization is not restricted to macrophages but represents a broader, energy‑dependent cellular process. Pharmacological inhibition of phagocytosis‑associated pathways markedly reduced intracellular fluorescence intensity, further supporting the involvement of active endocytic mechanisms.

To further evaluate cell‑type localization within a complex microenvironment, a three‑dimensional synovial organoid model was established [16]. Immunofluorescence staining demonstrated marked spatial overlap between CY5.5‑labeled RCEVs and CD11b⁺ macrophages (Figure S9A, B). Line‑scan fluorescence intensity analysis across representative regions showed highly coincident signal distributions between RCEVs and CD11b, with overlapping peak intensities along the scanned trajectories, supporting preferential macrophage localization. In contrast, co‑staining with fibroblast activation protein (FAP) revealed comparatively limited spatial coincidence between RCEVs and FAP⁺ fibroblast‑like cells (Figure S9C, D). Line‑scan analysis confirmed reduced signal overlap relative to CD11b⁺ regions, indicating that although RCEVs were detectable within fibroblast‑like cells, fluorescence enrichment was more prominent in macrophage populations within the synovial organoid.

Finally, in vivo fluorescence imaging demonstrated prolonged systemic retention of RCEVs‑CY5.5 compared with free dye (Fig. 3G, with gradual redistribution and clearance over 24 h. Ex vivo organ imaging confirmed differential accumulation profiles and predictable clearance kinetics.

Collectively, these results demonstrate that RCEVs undergo energy‑dependent internalization through multiple endocytic pathways, evade lysosomal degradation, associate with mitochondria, preferentially localize to macrophages within synovial tissue models relative to fibroblast‑like cells, and exhibit enhanced in vivo retention compared with free dye, supporting their function as bioactive nanocarriers for macrophage‑associated modulation.

### RCEVs@SPD attenuate pro‑inflammatory macrophage activation and promote anti‑inflammatory polarization

**Fig. 4:**
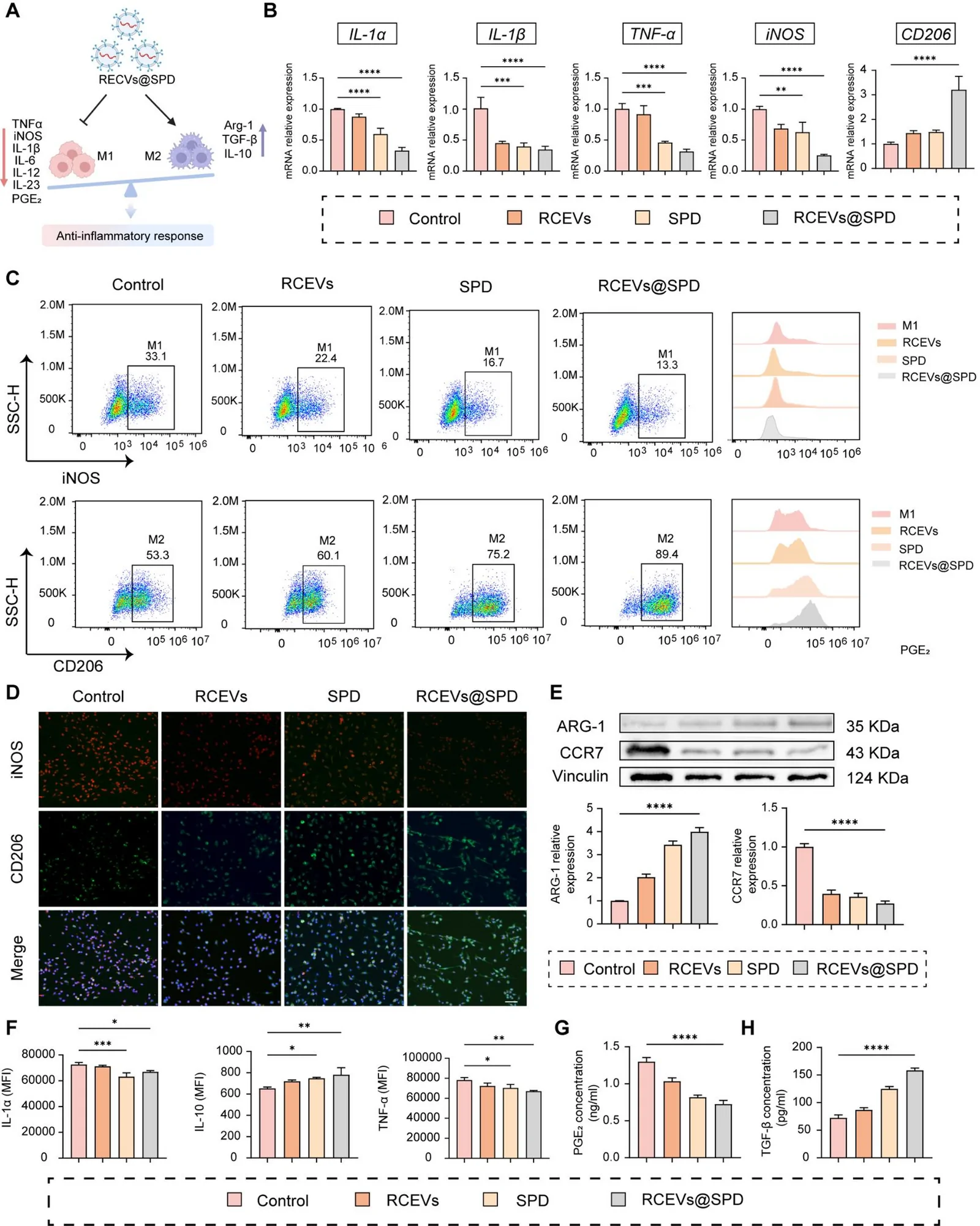
RCEVs@SPD reprogram primary BMDMs toward an anti‑inflammatory phenotype. (**A**) Schematic illustration showing that RCEVs@SPD suppress pro-inflammatory M1 polarization while promoting anti-inflammatory M2 polarization of macrophages. (**B**) Quantitative RT‑PCR analysis of M1‑associated genes and the M2 marker CD206 in BMDMs treated with Control, RCEVs, SPD, or RCEVs@SPD. (**C**) Flow cytometric analysis of iNOS⁺ (M1) and CD206⁺ (M2) macrophage populations under different treatments. Representative dot plots and quantitative analysis are shown. (**D**) Representative immunofluorescence images of primary BMDMs stained for iNOS (red) and CD206 (green), with nuclei counterstained using DAPI (blue). Scale bar = 100 μm. (**E**) Western blot analysis of ARG‑1 and CCR7 protein expression in treated primary BMDMs, with vinculin serving as the loading control. Densitometric quantification of protein levels is presented on the right. (**F**) Quantitative measurement of cytokines (IL‑1α, IL‑10, and TNF‑α) in primary BMDM culture supernatants using a multiplex bead‑based cytokine detection kit analyzed by flow cytometry. (**G**) Quantification of PGE_2_ levels in primary BMDM culture supernatants. (**H**) Quantitative analysis of TGF‑β levels in primary BMDM culture supernatants. Data are presented as mean ± SD (*n* = 3). Statistical significance was analyzed by one‑way ANOVA with appropriate post hoc tests. *P < 0.05, **P < 0.01, ***P < 0.001, ****P < 0.0001

To investigate whether RCEVs@SPD regulate macrophage activation, primary bone marrow-derived macrophages (BMDMs) were treated with RCEVs, SPD, or RCEVs@SPD (Fig. 4A). Macrophage polarization status was assessed at transcriptional, cellular, protein, and cytokine levels. Quantitative RT‑PCR analysis revealed that RCEVs@SPD markedly suppressed M1‑associated genes, including IL‑1α, IL‑1β, TNF‑α, and iNOS, compared with the Control group. In parallel, the M2 marker CD206 was significantly upregulated, indicating a transcriptional shift toward alternative polarization (Fig. 4B). Flow cytometric analysis further substantiated this phenotypic transition. The proportion of iNOS⁺ (M1) macrophages was progressively reduced following treatment and reached the lowest level in the RCEVs@SPD group (Fig. 4C). Conversely, CD206⁺ (M2) macrophages were markedly increased, consistent with enhanced anti‑inflammatory polarization. Representative dot plots and distribution histograms demonstrated a clear redistribution of macrophage subsets. Immunofluorescence staining confirmed reduced intracellular iNOS expression and elevated CD206 signal intensity in RCEVs@SPD‑treated BMDMs (Fig. 4D). At the protein level, Western blot analysis showed significantly increased ARG‑1 and decreased CCR7 expression compared with Control (Fig. 4E), further supporting suppression of classical activation and promotion of an alternative phenotype. Functional cytokine profiling revealed concordant remodeling of the inflammatory milieu. Multiplex bead‑based flow cytometric analysis demonstrated that RCEVs@SPD significantly decreased IL‑1α and TNF‑α secretion while enhancing IL‑10 production (Fig. 4F). In addition, the pro‑inflammatory lipid mediator prostaglandin E2 (PGE2) was reduced (Fig. 4G), whereas TGF‑β levels were significantly elevated in culture supernatants (Fig. 4H).

To comprehensively evaluate global cytokine modulation, multiplex cytokine profiling was performed. Heatmap analysis revealed an overall attenuation of pro‑inflammatory cytokine signatures in the RCEVs@SPD group compared with Control and single treatments (Figure S10A). Notably, key inflammatory amplifiers, including IL‑6 and GM‑CSF, were significantly reduced as quantified by mean fluorescence intensity (Figure S10B, C), indicating broader suppression of inflammatory network activation.

Collectively, these data demonstrate that RCEVs@SPD effectively reprogram primary macrophages by attenuating classical M1 activation while promoting anti‑inflammatory M2 polarization, accompanied by coordinated remodeling of cytokine production toward a pro‑resolving phenotype.

### RCEVs@SPD reprogram macrophage metabolism by suppressing glycolysis and restoring oxidative phosphorylation

**Fig. 5:**
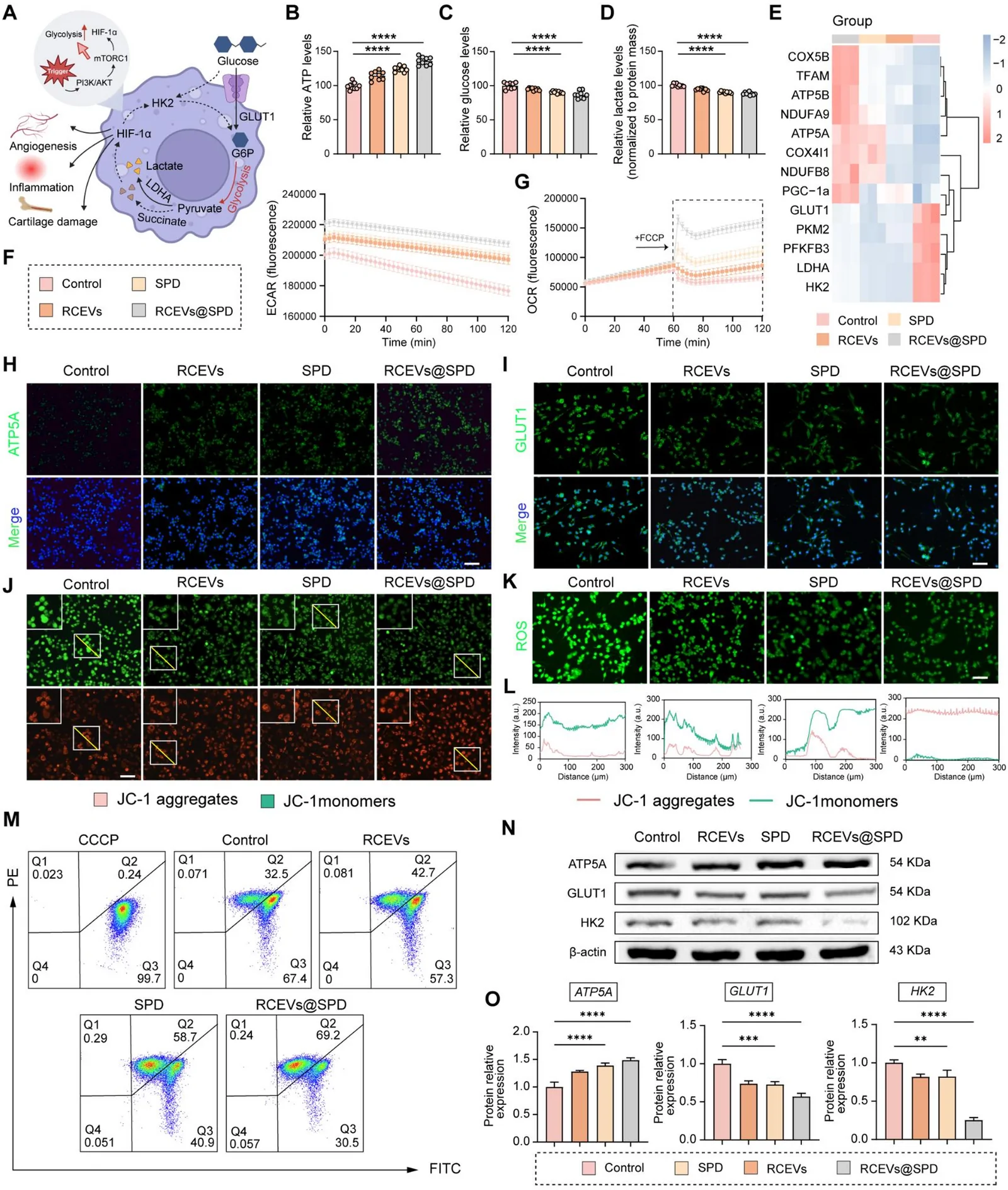
RCEVs@SPD remodel macrophage metabolic programming by suppressing glycolysis and enhancing mitochondrial function. (**A**) Schematic illustration of the proposed metabolic regulatory mechanism of RCEVs@SPD in macrophages. RCEVs@SPD inhibit PI3K/AKT–driven glycolysis while promoting mitochondrial oxidative phosphorylation, thereby shifting macrophages toward a metabolically quiescent anti‑inflammatory phenotype. (**B-D**) Quantitative analysis of intracellular ATP levels (B), glucose consumption (**C**), and lactate production normalized to total protein content (**D**) in macrophages treated with Control, RCEVs, SPD, or RCEVs@SPD. (**E**) Heatmap of metabolic gene expression profiles, including glycolysis‑related and mitochondrial oxidative phosphorylation-associated genes. Data are normalized and presented as Z‑scores. (**F**) ECAR measured using a commercial assay kit and quantified with a microplate reader, reflecting glycolytic activity under different treatments. (**G**) OCR determined using a commercial detection kit and analyzed by microplate reader. FCCP was added at the indicated time point to evaluate maximal respiratory capacity. (**H**) Immunofluorescence staining of ATP5A (green) in macrophages. Nuclei were counterstained with DAPI (blue). (**I**) Immunofluorescence staining of GLUT1 (green) in macrophages. Nuclei were counterstained with DAPI (blue). (**J**) JC‑1 staining for mitochondrial membrane potential (ΔΨm). Red fluorescence represents JC‑1 aggregates (polarized mitochondria), whereas green fluorescence indicates JC‑1 monomers (depolarized mitochondria). (**K**) Intracellular reactive oxygen species (ROS) levels detected by fluorescence staining. (**L**) Representative fluorescence intensity profiles of JC‑1 aggregates and monomers. (**M**) Flow cytometric analysis of mitochondrial membrane potential using JC‑1 staining. CCCP was used as a positive control for mitochondrial depolarization. (**N**) Western blot analysis of ATP5A, GLUT1, and HK2 protein expression, with β‑actin as the loading control. (**O**) Densitometric quantification of ATP5A, GLUT1, and HK2 protein levels. Data are presented as mean ± SD (*n* = 3). Statistical significance was analyzed by one‑way ANOVA with appropriate post hoc tests. **p < 0.01, ***p < 0.001, ****p < 0.0001

To determine whether RCEVs@SPD modulate macrophage metabolic programming, we systematically evaluated glycolytic activity and mitochondrial function. As illustrated in the proposed mechanism (Fig. 5A), RCEVs@SPD are hypothesized to inhibit PI3K/AKT-driven glycolysis while enhancing oxidative phosphorylation. Functionally, RCEVs@SPD significantly increased intracellular ATP levels compared with control and single‑component treatments (Fig. 5B). Meanwhile, glucose consumption and lactate production were markedly reduced (Fig. 5C, D), indicating suppression of glycolytic flux. Gene expression profiling further revealed coordinated metabolic remodeling. Glycolysis‑related genes, including GLUT1, HK2, PFKFB3, PKM2, and LDHA, were downregulated, whereas mitochondrial oxidative phosphorylation-associated genes, such as ATP5A, COX4I1, NDUFB8, TFAM, and PGC‑1α, were upregulated (Fig. 5E). These findings suggest a metabolic shift from aerobic glycolysis toward mitochondrial respiration. Consistently, extracellular acidification rate (ECAR), measured using a commercial detection kit, was significantly decreased following RCEVs@SPD treatment (Fig. 5F), while oxygen consumption rate (OCR) was markedly elevated (Fig. 5G), particularly after FCCP stimulation, indicating enhanced maximal respiratory capacity. At the protein level, immunofluorescence staining demonstrated increased ATP5A expression and reduced GLUT1 expression in RCEVs@SPD‑treated macrophages (Fig. 5H, I). Western blot analysis further confirmed upregulation of ATP5A and downregulation of GLUT1 and HK2 (Fig. 5N, O). JC‑1 staining revealed restoration of mitochondrial membrane potential, as indicated by an increased red/green fluorescence ratio (Fig. 5J-M), accompanied by a significant reduction in intracellular ROS levels (Fig. 5K), suggesting improved mitochondrial integrity and reduced oxidative stress.

To explore the signaling mechanisms underlying these metabolic alterations, we examined PI3K/AKT/AMPK pathway activation. RCEVs@SPD significantly decreased the phosphorylation levels of PI3K and AKT while enhancing AMPK phosphorylation, as quantified by normalization of phosphorylated proteins to their respective total protein levels (Figure S11A, B; S12). Importantly, pharmacological activation of PI3K signaling using 740Y‑P restored PI3K and AKT phosphorylation and partially reversed the inhibitory effects of RCEVs@SPD (Figure S11C, D; S13), indicating that metabolic reprogramming induced by RCEVs@SPD is associated with modulation of PI3K/AKT signaling.

Collectively, these results demonstrate that RCEVs@SPD suppress glycolytic metabolism and restore mitochondrial oxidative phosphorylation in macrophages, consistent with the proposed regulatory model, thereby promoting a metabolically quiescent anti‑inflammatory phenotype.

### RCEVs@SPD rewire the macrophage transcriptome toward oxidative metabolism

**Fig. 6:**
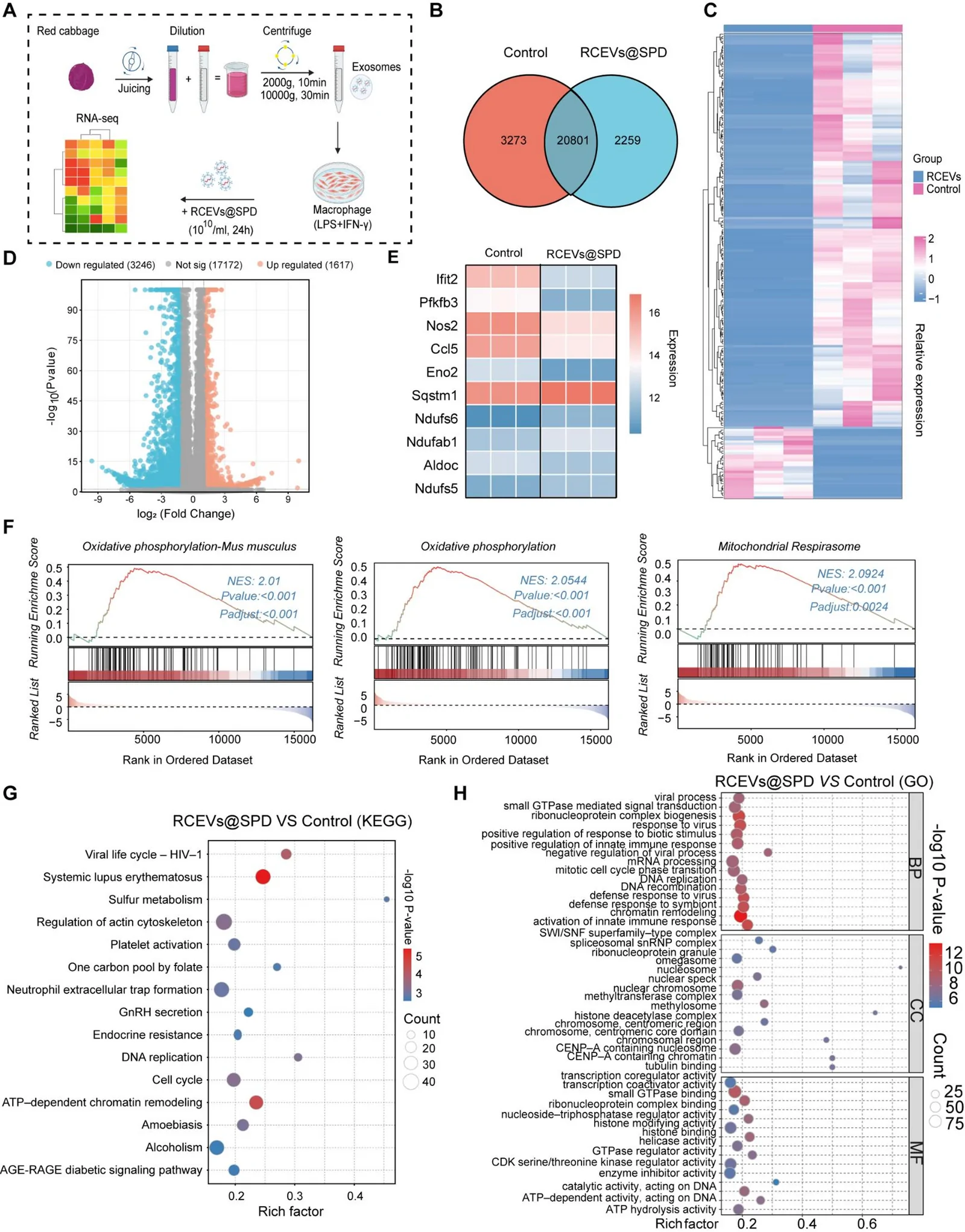
RCEVs@SPD rewires macrophage transcription toward oxidative metabolism. (**A**) Workflow: isolation of RCEVs, SPD loading (RCEVs@SPD), macrophage treatment, and bulk RNA-seq. (**B**) Venn diagram showing the overlap of detected genes between Control and RCEVs@SPD groups. (**C**) Heatmap of DEGs across samples (row-scaled counts), highlighting broad transcriptional remodeling by RCEVs@SPD. (**D**) Volcano plot of DEGs between RCEVs@SPD and Control (|log2FC| > 1 and FDR < 0.05); numbers in the plot indicate up- and down-regulated genes. (**E)** Targeted view of representative genes: glycolysis/inflammatory markers (Pfkfb3, Eno2, Aldoc, Nos2, Ccl5, Sqstm1) and mitochondrial complex-I subunits (Ndufs6, Ndufab1, Ndufs5), showing reduced glycolytic/inflammatory programs and increased respiratory chain components after RCEVs@SPD. (**F**) GSEA demonstrating significant positive enrichment of oxidative phosphorylation-associated pathways, including Oxidative phosphorylation (Mus musculus), Oxidative phosphorylation, and Mitochondrial respirasome, in the RCEVs@SPD group, indicating enhanced mitochondrial respiratory activity. (**G**) KEGG over-representation of DEGs; bubble size indicates gene count and color encodes -log10 (adjusted *p* value). (**H**) GO enrichment (BP/CC/MF) of DEGs, highlighting ATP synthesis-coupled electron transport, mitochondrial respiratory chain complex assembly, oxidoreductase activity, ribosomal/translation terms, and DNA repair/organization

To comprehensively characterize the transcriptional programs underlying RCEVs@SPD-induced metabolic reprogramming, bulk RNA sequencing was performed in macrophages treated with RCEVs@SPD or vehicle control (Fig. 6A). A Venn diagram showed substantial overlap in detected genes between the two groups, indicating comparable transcriptome coverage and data quality (Fig. 6B). Unsupervised hierarchical clustering of differentially expressed genes (DEGs) revealed a clear separation between Control and RCEVs@SPD-treated macrophages, demonstrating broad transcriptional remodeling upon RCEVs@SPD treatment (Fig. 6C). Differential expression analysis identified a large set of genes significantly altered by RCEVs@SPD (|log₂FC| > 1, FDR < 0.05), as visualized by volcano plot analysis (Fig. 6D). Targeted inspection of representative genes revealed that key glycolysis- and inflammation-associated genes, including Pfkfb3, Eno2, Aldoc, Nos2, Ccl5, and Sqstm1, were markedly downregulated in RCEVs@SPD-treated macrophages. In contrast, multiple mitochondrial respiratory chain complex I-related genes, such as Ndufs6, Ndufab1, and Ndufs5, were upregulated (Fig. 6E), indicating a transcriptional shift away from glycolytic and inflammatory programs toward mitochondrial respiration.

GSEA further supported this metabolic transition, revealing significant positive enrichment of oxidative phosphorylation-related pathways, including Oxidative phosphorylation (Mus musculus), Oxidative phosphorylation, and Mitochondrial respirasome, in the RCEVs@SPD group (Fig. 6F; S14). Consistently, KEGG pathway over-representation analysis revealed enrichment of metabolic and mitochondrial-related pathways among the DEGs (Fig. 6G; S15A). GO enrichment analysis across biological process (BP), cellular component (CC), and molecular function (MF) categories further highlighted terms associated with ATP synthesis-coupled electron transport, mitochondrial respiratory chain complex assembly, oxidoreductase activity, and translational machinery (Fig. 6H; S15B).

Collectively, these transcriptomic analyses demonstrate that RCEVs@SPD induce a coordinated reprogramming of the macrophage transcriptional landscape, characterized by suppression of glycolytic and inflammatory gene networks and enhancement of oxidative metabolic and mitochondrial respiratory programs.

### Untargeted metabolomics reveals global metabolic remodeling in LPS-stimulated macrophages after RCEVs@SPD treatment

To capture the small-molecule consequences of the transcriptomic shift toward oxidative metabolism, we performed LC-MS-based untargeted metabolomics in both ESI negative and positive modes. Data quality and feature coverage were robust across biological replicates, as indicated by highly consistent TIC profiles in negative/positive modes and dense RT-m/z feature distributions spanning broad chromatographic windows (Figure S16). Overall feature detection and annotation depth are summarized in Table S4: 29,071 MS¹ features were detected (negative 11,891; positive 17,180), including 1,118 MS²-confirmed features (negative 580; positive 538). Database matching yielded extensive candidate annotations (HMDB: 7,576 negative / 13,055 positive; KEGG: 4,741 negative / 8,009 positive), resulting in 20,685 annotated MS¹ features in total (7,606 negative; 13,079 positive) (Table S5). At the MS¹ level, the chemical space was dominated by lipid and lipid-like molecules, organic acids/derivatives, and organoheterocyclic compounds, with broad KEGG Level-2 functional coverage mainly mapping to amino-acid, lipid, and carbohydrate metabolism (Figure S17). Importantly, MS²-confirmed features showed high identification scores and coherent class-dependent RT-m/z distributions, supporting the reliability of downstream pathway inference; KEGG pathway evidence derived from MS² features further provided a higher-confidence layer for functional interpretation (Figure S18).

**Fig. 7:**
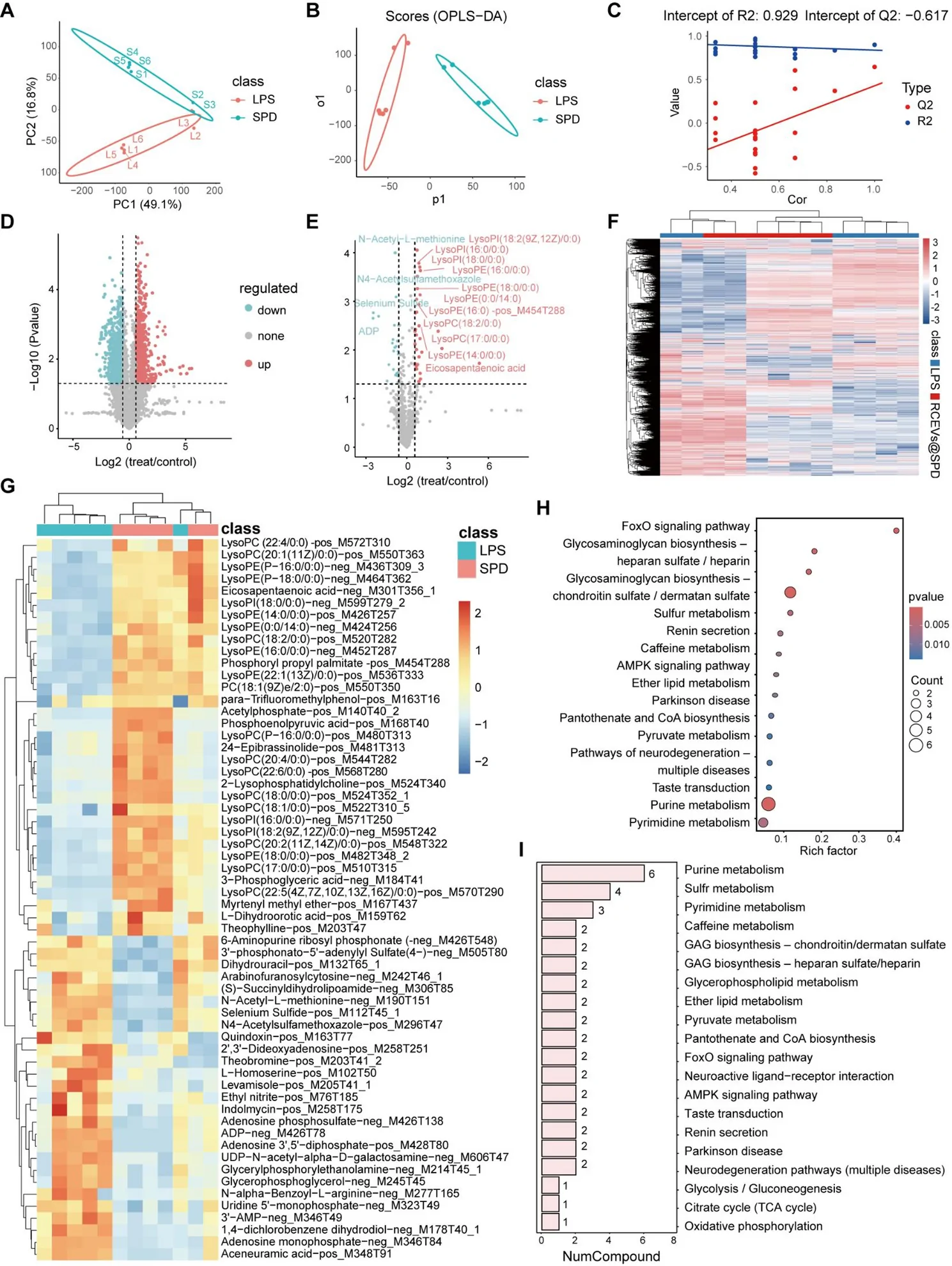
Global metabolomic profiling reveals extensive metabolic remodeling induced by RCEVs@SPD. (**A**) Principal component analysis (PCA) score plot showing global metabolic separation between LPS-stimulated macrophages and RCEVs@SPD-treated macrophages. (**B, C**) Orthogonal partial least squares discriminant analysis (OPLS-DA) score plot and corresponding permutation test validating the robustness and reliability of the model. (**D**) Volcano plot displaying significantly altered metabolic features between groups (fold change and statistical significance). (**E**) Volcano plot showing significantly altered metabolites with MS²-level annotation between LPS and RCEVs@SPD groups. (**F**) Hierarchical clustering heatmap of differential metabolites illustrating distinct metabolic signatures across samples. (**G**) Hierarchical clustering heatmap of MS²-confirmed differential metabolites, illustrating relative abundance patterns across LPS and RCEVs@SPD samples. Rows represent individual metabolites and columns represent biological replicates. Color scale indicates Z-score–normalized metabolite abundance. (**H**) KEGG pathway enrichment analysis based on MS²-supported differential metabolites, presented as a bubble plot. (**I**) Top enriched KEGG pathways derived from MS²-level metabolite annotations

Multivariate analyses demonstrated clear metabolic divergence between LPS and RCEVs@SPD groups. PCA score plots showed separation of the two groups, and OPLS-DA models further discriminated LPS from RCEVs@SPD with permutation testing supporting model validity (Fig. 7A-C). Differential analysis identified 1,769 significantly altered features across both ion modes, with directional changes summarized in Table S4 (negative: 339 down / 413 up; positive: 514 down / 503 up). These alterations were visualized by volcano plots and hierarchical clustering heatmaps, which revealed consistent within-group concordance and clear between-group separation (Fig. 7D).

To contextualize the altered metabolite landscape, KEGG functional classification of detected metabolites showed that changes were predominantly assigned to the “Metabolism” category, and Level-2 distributions concentrated on amino-acid metabolism, lipid metabolism, and carbohydrate metabolism (Figure S19D). KEGG enrichment of differential metabolites highlighted multiple metabolism-linked pathways, with enriched terms including central carbon and cofactor routes (e.g., pentose phosphate pathway and pantothenate & CoA biosynthesis) as well as amino-acid and nucleotide metabolism (e.g., glycine/serine/threonine metabolism, alanine/aspartate/glutamate metabolism, lysine degradation, pyrimidine metabolism and related pathways) (Figure S19C). In parallel, KEGG subcategory mapping and pathway-level summaries further supported broad remodeling across amino-acid, lipid, and carbohydrate metabolic modules (Figure S19A, B).

To strengthen biological interpretation using multi-level annotation, we next focused on differential metabolites with explicit MS²support and performed pathway enrichment based on MS²-identified features. Volcano visualization highlighted annotated lipid-related species among the significantly changed metabolites, and MS²-level clustering heatmaps demonstrated reproducible groupwise abundance patterns between LPS and RCEVs@SPD (Fig. 7E-G). KEGG classification of quantified metabolites (MS¹-level) and differential subsets (MS¹-level) consistently emphasized metabolism-dominated distributions across Level-1/Level-2 categories (Figure S20A, B).

Critically, MS²-level KEGG enrichment of differential metabolites identified signaling- and metabolism-connected pathways, including AMPK signaling and FoxO signaling, ether lipid metabolism, sulfur metabolism, pantothenate & CoA biosynthesis, purine/pyrimidine metabolism, and additional pathway terms derived from the MS²-supported differential set (Fig. 7H, I).

Together, these untargeted metabolomics results supported by stringent QC/coverage, annotation depth and chemical-class composition, MS² confidence and pathway evidence, and the global and MS²-focused differential/pathway analyses demonstrate that RCEVs@SPD induces a broad, reproducible metabolic rewiring in LPS-stimulated macrophages, providing metabolite-level support for a treatment-associated shift in core metabolic and signaling-linked pathways.

### RCEVs@SPD alleviate arthritis progression and restore joint homeostasis in CIA mice

**Fig. 8:**
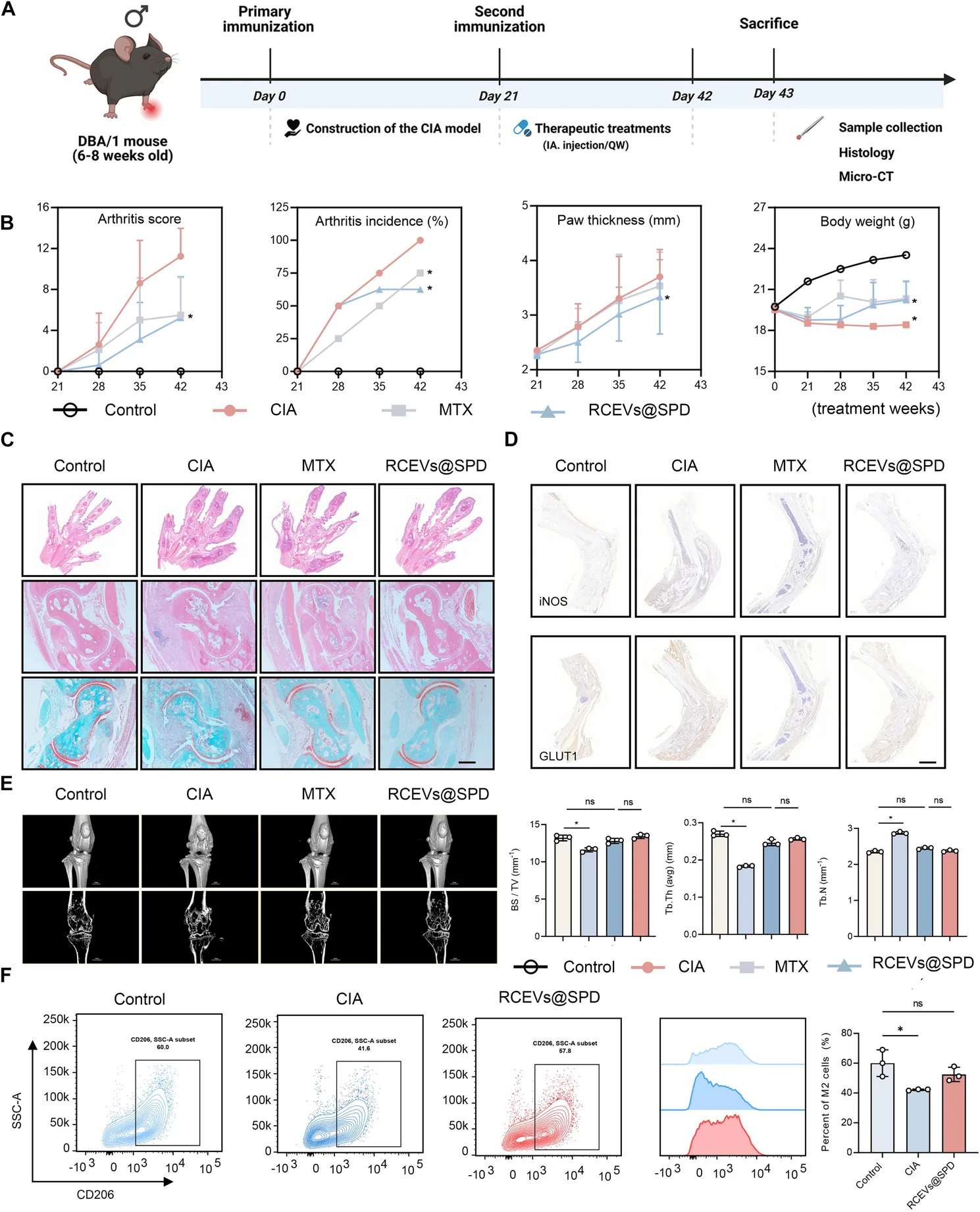
RCEVs@SPD alleviate arthritis progression, suppress synovial inflammation, and restore joint integrity in CIA mice. (**A**) Schematic illustration of the experimental design. (**B**) Longitudinal evaluation of arthritis progression, including arthritis score, arthritis incidence, paw thickness, and body weight throughout the treatment period. Data are presented as mean ± SEM. (**C**) Representative histological images of paws and ankle joints from Control, CIA, MTX, and RCEVs@SPD-treated mice, showing joint architecture, inflammatory cell infiltration, cartilage erosion, and bone destruction (H&E and Safranin O/Fast Green staining). (**D**) Representative immunohistochemical staining of ankle joint sections for the glycolysis-associated glucose transporter GLUT1 and the pro-inflammatory marker iNOS, illustrating metabolic and inflammatory modulation across different groups. (**E**) Representative three-dimensional micro‑CT reconstructions of ankle joints and corresponding quantitative analysis of bone structural parameters, including bone surface to tissue volume ratio (BS/TV), trabecular thickness (Tb.Th), and trabecular number (Tb.N). (**F**) Flow cytometric analysis of CD206 expression in joint-associated immune cells. Representative contour plots and histogram overlays are shown, with quantification of CD206⁺ (M2-like) macrophages on the right. Statistical significance was determined by one-way ANOVA with appropriate post hoc tests (*p < 0.05; ns, not significant)

The therapeutic efficacy of RCEVs@SPD was evaluated in a CIA mouse model (Fig. 8A). CIA mice developed progressive joint swelling and severe arthritis, as reflected by significantly elevated arthritis scores, increased paw thickness, and high disease incidence compared with healthy controls. Treatment with RCEVs@SPD markedly attenuated disease progression, significantly reducing arthritis scores and paw swelling throughout the treatment period (Fig. 8B). Body weight remained stable in the RCEVs@SPD group, indicating good systemic tolerance. Gross observation of paws further confirmed that RCEVs@SPD alleviated erythema and edema compared with untreated CIA mice (Figure S21A), accompanied by partial normalization of peripheral blood inflammatory parameters (Figure S21B).

Histopathological analysis revealed pronounced synovial hyperplasia, inflammatory cell infiltration, cartilage erosion, and bone destruction in CIA mice. In contrast, RCEVs@SPD treatment markedly reduced inflammatory infiltration and preserved joint architecture, as shown by H&E and Safranin O/Fast Green staining (Fig. 8C). Quantitative assessment demonstrated a significant reduction in intra-articular inflammatory cell area in the RCEVs@SPD group (Figure S22A), confirming effective suppression of synovial inflammation.

Given the pivotal role of metabolic reprogramming in inflammatory macrophage activation, we next examined glycolytic and pro-inflammatory markers in synovial tissues. Immunohistochemical staining showed elevated expression of GLUT1 and iNOS in CIA joints, indicating enhanced glycolysis and pro-inflammatory activation. RCEVs@SPD treatment significantly decreased the percentage of GLUT1- and iNOS-positive areas (Fig. 8D; Figure S22B, C), suggesting metabolic modulation and attenuation of inflammatory activation. Flow cytometric analysis further demonstrated an increased proportion of CD206⁺ macrophages in the RCEVs@SPD group (Fig. 8F), indicating a shift toward an anti-inflammatory M2-like phenotype. Consistently, intracellular cytokine analysis revealed marked reductions in IL‑1α, IL‑1β, IL‑6, and TNF‑α levels in joint immune cells following RCEVs@SPD treatment (Figure S23).

Micro‑CT analysis further demonstrated severe trabecular bone loss and structural deterioration in CIA mice. In contrast, RCEVs@SPD markedly preserved bone microarchitecture, as evidenced by improved bone surface to tissue volume ratio (BS/TV), increased trabecular thickness (Tb.Th), and higher trabecular number (Tb.N) (Fig. 8E), indicating effective protection against inflammatory bone destruction.

Finally, biosafety evaluation showed no obvious pathological abnormalities in major organs, including heart, liver, spleen, lung, and kidney (Figure S24). Serum biochemical analysis revealed no significant alterations in hepatic (ALT, AST, γ‑GT) or renal (UA, CREA, UREA) function indicators among groups (Figure S25), supporting the favorable safety profile of RCEVs@SPD.

Collectively, these results demonstrate that RCEVs@SPD effectively suppress synovial inflammation, modulate metabolic and immune responses, protect bone integrity, and exhibit good systemic biosafety in CIA mice.

### Macrophages are required for the therapeutic efficacy of RCEVs@SPD in CIA mice

**Fig. 9:**
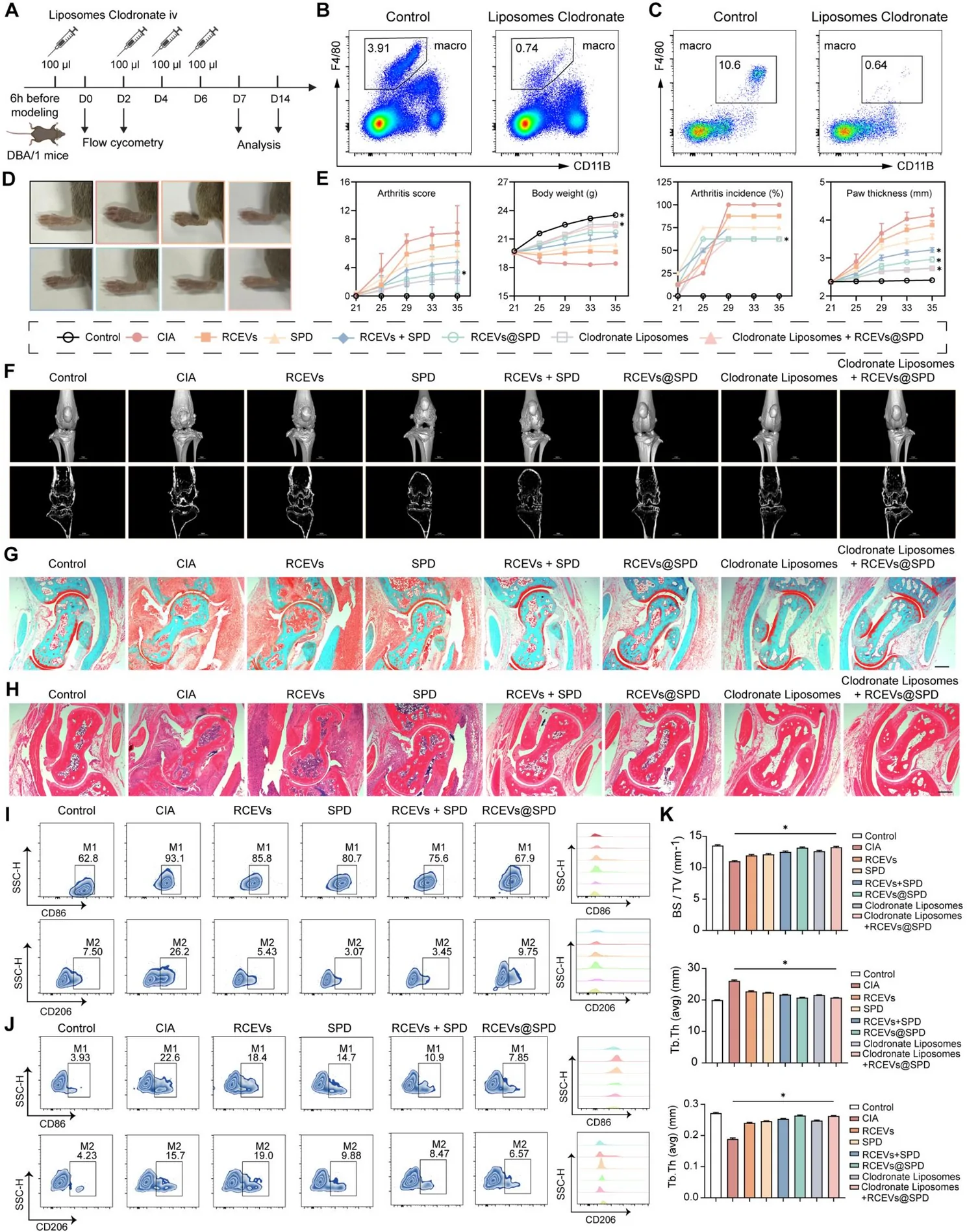
Macrophages are required for the therapeutic efficacy of RCEVs@SPD in CIA mice. (**A**) Schematic illustration of the experimental design. Collagen‑induced arthritis (CIA) was established in DBA/1 mice. Clodronate liposomes (100 µL, i.v.) were administered at the indicated time points to deplete macrophages. Flow cytometry was performed to verify depletion efficiency prior to therapeutic evaluation. (**B**) Flow cytometric analysis of CD11b⁺F4/80⁺ macrophages in the spleen, confirming effective systemic macrophage depletion following clodronate liposome administration. (**C**) Flow cytometric analysis of CD11b⁺F4/80⁺ macrophages in peritoneal lavage fluid (ascites), further validating macrophage depletion efficiency. (**D**) Representative photographs of hind paws from each experimental group. (**E**) Clinical assessment of arthritis progression, including arthritis score, body weight, arthritis incidence, and paw thickness. (**F**) Representative micro‑CT images of ankle joints demonstrating bone erosion and structural alterations. (**G**) Safranin O-Fast Green staining of ankle joint sections to evaluate cartilage integrity. (**H**) Hematoxylin and eosin (H&E) staining of joint sections showing synovial hyperplasia and inflammatory infiltration. (**I**) Flow cytometric analysis of macrophage polarization in synovial tissue, showing representative contour plots and quantitative proportions of M1 (CD86⁺) and M2 (CD206⁺) macrophages among CD11b⁺F4/80⁺ cells. (**J**) Flow cytometric analysis of macrophage polarization in peritoneal lavage fluid, demonstrating alterations in M1 (CD86⁺) and M2 (CD206⁺) macrophage subsets. (**K**) Quantitative analysis of micro‑CT parameters, including bone surface to tissue volume ratio (BS/TV), trabecular thickness (Tb.Th), and related structural indices. Statistical significance was determined by one‑way ANOVA with appropriate post hoc tests. *p < 0.05

To determine whether macrophages are indispensable mediators of RCEVs@SPD therapy, systemic macrophage depletion was achieved using clodronate liposomes in CIA mice according to the experimental scheme shown in Fig. 9A. Flow cytometric analysis confirmed efficient depletion of CD11b⁺F4/80⁺ macrophages in both the spleen and peritoneal lavage fluid (Fig. 9B, C), validating the effectiveness of the depletion strategy prior to therapeutic assessment.

As expected, CIA mice exhibited progressive joint swelling and severe arthritis. Treatment with RCEVs@SPD markedly alleviated clinical symptoms, whereas clodronate-mediated macrophage depletion alone also attenuated disease severity, consistent with the established pathogenic role of inflammatory macrophages in CIA. Notably, when RCEVs@SPD was administered following macrophage depletion, no further improvement beyond that observed in the clodronate-treated group was detected. Representative paw images and clinical evaluations demonstrated that the reductions in arthritis score, paw thickness, and disease incidence achieved by RCEVs@SPD were largely abolished in macrophage-depleted mice (Fig. 9D, E), while body weight remained comparable among groups. Micro‑CT analysis further supported this observation. Although RCEVs@SPD preserved trabecular bone architecture in CIA mice, and clodronate alone mitigated bone erosion, the combined treatment failed to confer additional structural protection beyond macrophage depletion alone (Fig. 9F, K). Histological examination by Safranin O/Fast Green and H&E staining revealed that the cartilage-preserving and anti-inflammatory effects of RCEVs@SPD were markedly attenuated in the absence of macrophages (Fig. 9G–H), indicating that its joint-protective efficacy depends on macrophage presence.

To further investigate immune modulation, macrophage polarization was analyzed in synovial tissues and peritoneal lavage fluid. In CIA mice, a predominance of CD86⁺ (M1-like) macrophages was observed, whereas RCEVs@SPD significantly reduced M1 proportions and increased CD206⁺ (M2-like) macrophages. However, following macrophage depletion, this polarization shift was no longer evident (Fig. 9I–J), confirming that the immunomodulatory effects of RCEVs@SPD require intact macrophage populations.

Collectively, although macrophage depletion alone partially ameliorated CIA pathology, it eliminated the additional therapeutic benefit conferred by RCEVs@SPD. These findings demonstrate that macrophages are essential cellular mediators of RCEVs@SPD-driven immunomodulation and joint protection in CIA mice.

## Discussion

Rheumatoid arthritis (RA) is increasingly recognized as a disease sustained by the interplay between dysregulated immune signaling and maladaptive cellular metabolism [6, 26–28]. In particular, macrophages constitute a dominant immune population within the inflamed synovium and orchestrate cytokine networks, stromal activation, and tissue destruction through metabolically driven inflammatory programs [29, 30]. Accumulating evidence indicates that pro-inflammatory macrophages rely on aerobic glycolysis, whereas anti-inflammatory and tissue-repairing phenotypes are supported by intact mitochondrial oxidative phosphorylation (OXPHOS), positioning immunometabolism as an actionable upstream determinant of inflammatory fate rather than a mere downstream consequence [31–33].

A key finding of this work is that RCEVs@SPD induces a coherent, system-level metabolic shift that extends beyond classical fatty acid oxidation-centered models of M2 polarization. Integrated untargeted metabolomics revealed extensive remodeling across central carbon metabolism, amino-acid metabolism, lipid metabolism, and cofactor biosynthesis, converging on enhanced mitochondrial respiration and redox balance. Enrichment of pantothenate and CoA biosynthesis, vitamin B6 metabolism, and the pentose phosphate pathway suggests increased provision of metabolic cofactors and reducing equivalents required for efficient OXPHOS. In parallel, prominent lysophospholipid and ether lipid remodeling indicates adaptive membrane reorganization associated with mitochondrial function and anti-inflammatory macrophage phenotypes [34].

Notably, we identified significant enrichment of sulfur metabolism and purine/pyrimidine metabolic pathways, pointing to a non-canonical mode of metabolic rewiring during macrophage polarization. Sulfur-containing metabolites, including cysteine- and glutathione-derived persulfides, have been shown to modulate intracellular redox tone and suppress pro-inflammatory gene expression, thereby promoting M2-like phenotypes under oxidative stress conditions [35, 36]. Concurrently, enhanced purine metabolism-particularly pathways governing NAD⁺ and ATP turnover-plays a pivotal role in macrophage energy homeostasis and polarization, reflecting increased mitochondrial respiratory demand and biosynthetic activity [37, 38]. These findings are consistent with recent studies demonstrating that metabolic intermediates beyond FAO, such as α-ketoglutarate, fumarate, and NAD⁺, act as active immunometabolic regulators that shape macrophage inflammatory fate.

At the signaling level, our multi-omics data support a model in which RCEVs@SPD engage conserved metabolic signaling axes to orchestrate this bioenergetic transition. Suppression of PI3K-AKT-associated glycolytic reinforcement, together with activation of AMPK- and FoxO-dependent programs, is consistent with the observed shift toward OXPHOS and mitochondrial fitness. Transcriptomic upregulation of mitochondrial electron transport chain components further corroborates enhanced respiratory capacity, a hallmark of anti-inflammatory macrophage states. Importantly, these signaling changes integrate with sulfur- and purine-linked metabolic rewiring, suggesting that spermidine functions not merely as a metabolic enhancer but as a redox- and nucleotide-sensitive immunometabolic regulator.

The delivery modality itself represents a critical determinant of therapeutic efficacy. Free SPD is limited by rapid degradation and insufficient intracellular accumulation [39]. In contrast, plant-derived extracellular vesicles serve as natural, biocompatible nanocarriers that protect cargo molecules while promoting efficient uptake by immune cells. Beyond passive delivery, plant vesicles possess intrinsic lipid and metabolite compositions that may synergize with spermidine to reinforce mitochondrial and redox-centered metabolic programs, consistent with emerging concepts of cross-kingdom vesicle-mediated communication [40]. Growing evidence suggests that PDEVs are not merely inert carriers but active biological entities capable of transferring bioactive cargos-including small RNAs, lipids, proteins, and metabolites-into mammalian recipient cells, thereby modulating inflammatory signaling and metabolic pathways [41]. Plant small RNAs exhibit enhanced stability due to 3′ terminal 2′-O-methylation, which may facilitate their persistence under physiological conditions and support functional interspecies communication. Moreover, recent studies indicate that PDEVs can influence immune cell polarization, redox balance, and mitochondrial activity in mammalian systems, although mechanistic validation and in vivo biodistribution dynamics remain areas of active investigation [42]. Within this evolving framework, our findings support the concept that RCEVs act as bioactive nanovesicles capable of engaging conserved endocytic and metabolic programs across kingdoms, thereby extending the paradigm of plant-mammalian molecular communication into the context of immunometabolic regulation in RA. This biomimetic strategy aligns cargo, carrier, and metabolic mechanism to achieve precise immunometabolic control.

The in vivo efficacy of RCEVs@SPD in CIA underscores the translational potential of macrophage‑targeted metabolic reprogramming. Attenuation of synovial inflammation, preservation of cartilage and bone integrity, and a shift toward anti‑inflammatory macrophage phenotypes collectively indicate meaningful disease modification, achieved without overt systemic toxicity. Macrophage‑depletion experiments further provide causal evidence for this mechanism. Although clodronate-mediated depletion partially alleviated arthritis, RCEVs@SPD failed to confer additional clinical or structural protection in macrophage‑depleted mice, and the CD86⁺‑to‑CD206⁺ polarization shift was abolished. These findings demonstrate that intact macrophage populations are indispensable for RCEVs@SPD efficacy, arguing against a macrophage‑independent mode of action.

Importantly, this distinction highlights the difference between macrophage elimination and functional re‑education. While depletion reduces inflammatory burden, broad macrophage ablation is not therapeutically sustainable given their roles in host defense and tissue repair. In contrast, RCEVs@SPD preserves macrophage populations while reshaping their metabolic and functional states, supporting macrophage metabolism as a rational disease‑modifying target in inflammatory arthritis.

Several limitations warrant consideration. Although this study focuses on macrophage-centered metabolic reprogramming, the RA synovial microenvironment comprises multiple interacting cell types, including fibroblast-like synoviocytes and adaptive immune cells, which also exhibit profound metabolic dysregulation. Whether RCEVs@SPD directly modulates redox and nucleotide metabolism in these populations remains to be determined. In addition, although macrophage-depletion experiments provide functional evidence that macrophages are required for the therapeutic efficacy of RCEVs@SPD, the clodronate liposome strategy induces broad systemic depletion of phagocytic macrophage populations and cannot discriminate the relative contributions of distinct macrophage subsets, such as synovial tissue-resident macrophages, infiltrating monocyte-derived macrophages, splenic macrophages, or peritoneal macrophages. Moreover, because macrophage depletion alone partially attenuated CIA severity, this model supports macrophage dependence but does not fully define which macrophage population is directly targeted by RCEVs@SPD or whether local vesicle uptake within the inflamed joint is essential for therapeutic efficacy. Future studies using macrophage subset-specific depletion, lineage tracing, adoptive transfer or rescue approaches, in vivo vesicle tracking, and single-cell multi-omics analyses will be valuable for resolving the spatial and functional heterogeneity of macrophage responses to RCEVs@SPD. Moreover, while PI3K-AKT and AMPK/FoxO pathways emerge as key regulatory nodes, causal links between spermidine delivery, sulfur/purine metabolism, and downstream signaling require further validation using genetic or pharmacological approaches. Finally, long-term biodistribution, pharmacokinetics, and dosing strategies for plant-derived vesicles warrant systematic evaluation to facilitate clinical translation.

In conclusion, this study expands the conceptual framework of macrophage immunometabolism in RA by identifying a redox- and nucleotide-centered mode of metabolic reprogramming driven by plant vesicle-mediated spermidine delivery. By coupling a naturally occurring polyamine with a scalable, biocompatible carrier, RCEVs@SPD restore mitochondrial bioenergetic balance, suppress glycolysis-driven inflammation, and ameliorate joint pathology. The macrophage-depletion findings further support macrophages as indispensable cellular mediators of this therapeutic effect, reinforcing the rationale for macrophage-targeted metabolic reprogramming as a potentially non-immunosuppressive therapeutic strategy for RA and related chronic inflammatory diseases.

## Conclusion

In conclusion, this study establishes an edible plant-derived extracellular vesicle-based strategy for immunometabolic intervention in RA. By engineering RCEVs@SPD, we demonstrate that macrophage-centered metabolic reprogramming shifts macrophage bioenergetics from glycolysis-dominated inflammatory activation toward mitochondrial oxidative phosphorylation, thereby suppressing pro-inflammatory programs and promoting an immunoregulatory phenotype. Integrated functional assays, transcriptomic profiling, and untargeted metabolomics reveal coordinated remodeling of inflammatory pathways and metabolic networks underlying this bioenergetic transition. Importantly, these macrophage-directed effects translated into robust therapeutic efficacy in vivo, reducing synovial inflammation and joint destruction in the CIA model while maintaining a favorable systemic safety profile. The loss of additional therapeutic benefit following macrophage depletion further identifies macrophages as indispensable cellular mediators of RCEVs@SPD-driven immunomodulation and joint protection. Collectively, our findings position macrophage immunometabolism as an actionable upstream node in RA pathogenesis and establish edible plant-derived extracellular vesicles as a scalable, biocompatible platform for metabolism-oriented and potentially non-immunosuppressive immunotherapy.

## Supplementary Information

Below is the link to the electronic supplementary material.

Supplementary Material 1

## Data Availability

Data will be made available on request.
