# Supplementary material for: Plant-derived extracellular vesicles deliver spermidine to reprogram macrophage metabolism and ameliorate rheumatoid arthritis
Source: J Nanobiotechnology. 2026 Jul 26;24:974. doi: 10.1186/s12951-026-04836-x (PMC13685380; doi:10.1186/s12951-026-04836-x)
Supplement: Supplementary file 1 [file 12951_2026_4836_MOESM1_ESM.docx]

**Plant-derived extracellular vesicles deliver spermidine to reprogram macrophage metabolism and ameliorate rheumatoid arthritis**

Xiaocheng Wang ^a, b, c,^ ^#^, Jiaxin He ^a, b, c, #^, Yixiang Hong ^d^, Xingyue Zeng ^d^, Min Li ^a, b, c,^ Shixiong Cao ^d^, Renge Liang ^a, b, c^, Gengmin Zhou ^a, b, c^, Qingwen Wang ^a, b, c, *^

^a^ Department of Rheumatism and Immunology, Peking University Shenzhen Hospital, Shenzhen 518036, China

^b^ Institute of Immunology and Inflammatory Diseases, Shenzhen Peking University-The Hong Kong University of Science and Technology Medical Center, Shenzhen 518036, China

^c^ Shenzhen Key Laboratory of Inflammatory and Immunology Diseases, Shenzhen 518036, China

^d^ Department of Rheumatology & Immunology, Beijing Key Laboratory for Rheumatism Mechanism and Immune Diagnosis (BZ0135), Peking University People's Hospital, Beijing, 100044, China

^#^ These authors contributed equally to this work.

**Qingwen Wang:** Department of Rheumatism and Immunology, Peking University Shenzhen Hospital, Shenzhen, 518036, China. Email: [wangqingwen@pkuszh.com](mailto:wangqingwen@pkuszh.com)

**Experimental section**

**WGCNA-based network construction and enrichment analysis of the GSE10500 dataset**

Weighted gene co‑expression network analysis (WGCNA) was conducted on transcriptomic data from the GSE10500 dataset using the R package WGCNA. Following log2 transformation, the 3,000 most variable probes were retained to construct an unsigned co‑expression network based on Pearson correlation. The soft‑thresholding power was selected according to the scale‑free topology criterion, and gene modules were detected with the blockwiseModules function (minModuleSize = 30; mergeCutHeight = 0.25). Module eigengenes were computed, and network topology was visualized using the ggNetView package. Differential expression between RA‑like and control samples was assessed using limma, with significance defined as adjusted 𝑃 < 0.05 (Benjamini–Hochberg FDR). Heatmaps were generated with pheatmap from Z‑score normalized expression values. Gene set enrichment analysis (GSEA) was performed using a pre‑ranked approach implemented in clusterProfiler, with genes ranked by log_2_ fold change; a custom‑curated gene set collection integrating Hallmark and KEGG annotations was employed to interrogate metabolism‑related pathways. GSEA enrichment curves and ranked‑gene distributions were visualized using enrichplot and GseaVis. GO enrichment analysis of differentially expressed genes was conducted with clusterProfiler, applying Benjamini-Hochberg correction for multiple testing. Relationships between enriched pathways and their contributing genes were depicted as Sankey diagrams generated with ggsankey.

**Cell culture**

RAW264.7 murine macrophages (TCM13, Chinese Academy of Sciences Cell Bank) were cultured in the designated complete medium (Servicebio, GZ20020-500ML). Cells were maintained at 37 °C in a humidified incubator with 5% CO₂. To minimize potential interference from endotoxin contamination in exosome preparations, Polymyxin B Sulfate (MCE, HY‑A0248) was added when necessary to neutralize residual endotoxin during cell‑based assays.

**Isolation of red-cabbage-derived extracellular vesicles**

Fresh red cabbage was thoroughly washed with sterile water and mechanically cut into approximately 1cm³ pieces. The minced material was homogenized using a commercial juicer and subsequently resuspended in ice-cold phosphate-buffered saline (PBS, 1×, pH 7.4). To maximize vesicle yield, the homogenization-resuspension procedure was repeated twice, and the resulting supernatants were pooled. The combined extract was subjected to a series of refrigerated centrifugation steps. Initial clarification was performed at 10,000 × g for 30 min at 4 °C to remove coarse particulates. The supernatant was then centrifuged at 2,000 × g for 10 min at 4 °C to eliminate residual cellular debris, followed by an additional centrifugation at 10,000 × g for 30 min at 4 °C to deplete large extracellular vesicles. The clarified supernatant was transferred into polycarbonate ultracentrifuge tubes for high-speed isolation. Extracellular vesicles were pelleted by ultracentrifugation at 110,000 × g for 75 min at 4 °C using an Optima™ L-90K ultracentrifuge (Beckman Coulter, USA) equipped with a Type 70 Ti fixed-angle rotor. The pellet was gently resuspended in 1 mL of sterile PBS and passed through a 0.22-μm PVDF membrane to remove residual particulates. A final purification step using identical ultracentrifugation parameters (110,000 × g, 75 min, 4 °C) was performed to ensure vesicle purity. Purified vesicles were reconstituted in 1× PBS, aliquoted into sterile cryovials under aseptic conditions, and stored at -80 °C until further use.

**Endotoxin quantification**

Endotoxin levels in exosome preparations were determined using a Limulus Amebocyte Lysate (LAL) kinetic turbidimetric assay (Endotoxin Detection Kit, Beyotime, C0271S). All procedures were performed with endotoxin‑free consumables. Exosome samples were thawed on ice and diluted in endotoxin‑free PBS to fall within the linear detection range. A standard curve was prepared using reference endotoxin standards (0.01-1.0 EU/mL). Aliquots of standards and samples (in duplicate) were mixed with equal volumes of LAL reagent in a pre‑warmed 96‑well endotoxin‑free plate and incubated at 37 °C in a kinetic microplate reader. The increase in turbidity was continuously recorded, and endotoxin concentrations were calculated from the time‑to‑threshold values using the instrument’s software. Spike‑and‑recovery controls were included to assess matrix interference, and results were accepted when recovery fell within 50-200%. Endotoxin levels were expressed as EU/mL, and samples with values below 0.5 EU/mL were considered acceptable for subsequent biological experiments.

**Exosome nanoparticle tracking analysis**

Purified exosome samples were retrieved from -80°C storage and thawed at 25°C in a temperature-controlled water bath, followed by immediate transfer to ice-cooled racks to maintain vesicle integrity. Prior to analysis, aliquots were diluted in sterile 1× phosphate-buffered saline (PBS, pH 7.4) to achieve optimal particle concentrations (10⁷-10⁹ particles/mL) within the instrument's detection range. Quantitative characterization was performed using a ZetaVIEW® S/N 22-756 nanoparticle tracking analyzer (NTA, ParticleMetrix GmbH, Germany) equipped with a 488 nm laser module. Samples were loaded into sterile polystyrene cuvettes and analyzed at 25°C under standardized measurement conditions. Triplicate recordings of 60-second tracking cycles were acquired for each sample, with inter-cycle rinsing using 0.22 μm-filtered PBS to prevent cross-contamination. Particle concentration and size distribution profiles were derived from Brownian motion trajectories using the Stokes-Einstein equation, with results expressed as mean ± standard deviation of triplicate measurements.

**Exosome morphological characterization by** **transmission electron microscopy**

Purified exosome samples were applied to glow-discharged carbon-coated copper grids (300 mesh) by depositing 5 μL aliquots followed by 5 min adsorption at ambient temperature. Excess liquid was carefully removed using sterile filter paper applied to the grid periphery. Negative staining was subsequently performed with 2% (w/v) uranyl acetate (pH 4.5) for 1 min at room temperature, after which residual stain was blotted following identical protocol. Grids were air-dried for 20 min in a desiccated chamber prior to imaging. High-resolution micrographs were acquired using a Tecnai G2 Spirit BioTwin transmission electron microscope (TEM, Thermo Fisher Scientific, USA) operated at 80 kV accelerating voltage. Image capture was performed with an Eagle™ 4k CCD camera under low-dose conditions (<5 e⁻/Å²), with magnifications calibrated using a cross-line grating replica (2160 lines/mm). Three independent preparations were analyzed per experimental group, with representative images selected based on structural integrity and staining uniformity. Scale bar annotations and contrast optimization were performed using Gatan DigitalMicrograph® software (v3.4.1) without altering original image data.

**Extracellular vesicle purity determination**

Extracellular vesicle (EV) samples were diluted appropriately. A total of 45 μL of EV suspension was mixed with 5 μL of 10% Triton X‑100 (final concentration = 1%), vortexed gently, and incubated on ice for 1 h to ensure complete membrane disruption. Particle size and concentration were measured before and after disruption using a nanoparticle tracking analyzer (NTA). Purity was calculated as:

Purity(%) = (1−𝐶_after_ / 𝐶_before_)×100%.

where 𝐶_before_ and 𝐶_after_ represent particle concentrations (Particles/mL) before and after Triton X‑100 treatment. The remaining particles after disruption indicate non‑vesicular impurities; higher purity reflects better EV isolation quality.

**Preparation of SPD-loaded red-cabbage extracellular vesicles (RCEVs@SPD)**

RCEVs were mixed with SPD in sterile microcentrifuge tubes and gently vortexed to ensure homogeneous dispersion. The mixture was placed on ice, and probe sonication was performed at 120 W using a pulse mode (3 s on, 3 s off) for a total of 60 cycles. To facilitate membrane recovery and vesicle stabilization after sonication, the suspension was subsequently incubated at 37 °C on a shaking platform for 2 h. To remove unencapsulated spermidine, the resulting suspension was purified using centrifugal ultrafiltration units with a 10 kDa molecular weight cut-off (MWCO). Given that SPD is a small molecule (145.25 Da), it readily passes through the ultrafiltration membrane, whereas extracellular vesicles (typically 50–200 nm in diameter) are retained. The samples were washed multiple times with phosphate-buffered saline (PBS) during ultrafiltration to ensure effective removal of free SPD. The retained vesicle fraction (RCEVs@SPD) was collected for subsequent characterization and biological experiments.

**Determination of encapsulation efficiency and drug loading content**

The concentration of SPD was quantified using UV-visible spectrophotometry. A standard calibration curve was established by measuring the absorbance of SPD solutions at its characteristic absorption wavelength (205 nm) over a defined concentration range (0.1–0.3 mg/mL). Linear regression analysis yielded a correlation coefficient of R^2^ = 0.9983, indicating excellent linearity. The amount of unencapsulated SPD in the filtrate was determined using the established standard curve. The amount of encapsulated SPD was calculated by subtracting the free SPD from the total SPD initially added. Encapsulation efficiency (EE%) and drug loading content (DLC%) were calculated using the following equations:

EE (%) = (weight of SPD encapsulated in RCEVs / total weight of SPD added) × 100%

DLC (%) = (weight of SPD encapsulated in RCEVs / total weight of RCEVs@SPD) × 100%

All measurements were performed in triplicate to ensure reproducibility. Purified RCEVs@SPD were either used immediately or stored at −80 °C for further experiments.

**Cellular Uptake and Retention**

The uptake and retention of CY5.5‑labeled RCEVs in MH7A and RAW 264.7 cells were evaluated. Both cell types were seeded onto glass coverslips and subsequently incubated with CY5.5‑RCEVs. Nuclei were counterstained with DAPI, and F‑actin filaments were visualized using an F‑actin staining kit. A fluorescence upright microscope was used to examine the intracellular colocalization and internalization of RCEVs at various time points.

**Endocytosis Mechanism of Plant-Derived Exosomes**

The endocytic mechanism of RCEVs was evaluated based on previously reported methods [1, 2]. RAW 264.7 cells were polarized to an M1 phenotype by stimulation with 100 ng mL⁻¹ LPS. M1 macrophages were pretreated for 1 h with specific endocytosis inhibitors, including M‑β‑CD (5 × 10⁻⁶ M), filipin (5 µg mL⁻¹), chlorpromazine (10 µg mL⁻¹), and AMH (3 × 10⁻³ M). CY5.5‑labeled RCEVs were then added for co‑incubation. Cellular images were acquired using an upright fluorescence microscope.

**CCK8 assay**

Cell viability was assessed using the CCK-8 assay. RAW264.7 cells were seeded in 96-well plates at 8,000 cells/well in 200 µL complete DMEM and allowed to adhere overnight. Cells were treated with RCEVs at 1×10^11^ particles /ml or 2×10^11^ particles /ml particles per well. Parallel plates were designated for day 1, 2, and 3 readouts. At each time point, CCK-8 reagent (APExBIO Corporation, USA) was added and incubated for 1-2 h at 37°C. Absorbance at 450 nm was measured using a microplate reader (Synergy H1, BioTek Instruments, USA).

**Flow cytometry analysis**

Bone marrow cells were harvested from C57BL/6 mice, filtered through 70 μm strainers, and differentiated into bone marrow-derived macrophages (BMDMs) for 7 days in complete media supplemented with recombinant mouse M-CSF (50 ng/mL). Pro-inflammatory polarization was induced with LPS (100 ng/mL) and IFN-γ (20 ng/mL) for 24 h. Cells were subsequently treated with RCEVs@SPD for 24 h. For flow cytometry, cells were collected, blocked with anti-mouse CD16/32, stained with viability dye and fluorophore-conjugated antibodies against F4/80, CD11b, CD86, and acquired on a flow cytometer.

**Cytokine Assay**

In BMDM cells, a pro-inflammatory phenotype was induced by adding LPS and IFN-γ. Subsequently, RCEVs@SPD was added at a concentration of 10^11^ particles/ml, and the cells were treated for 24 hours. After treatment, the cell supernatants were collected for analysis. Cytokine levels were measured using the LEGENDplex™ MU Inflam panel (13-plex)w/VbP cytokine detection kit (Biolegend, 740446), and all procedures were strictly performed according to the manufacturer's instructions. The supernatants were then analyzed on a CytoFLEX LX flow cytometer (Beckman Coulter, USA) for cytokine quantification.

**Transcriptomic sequencing**

RAW264.7 cells were stimulated with LPS and IFN-γ and subsequently treated with RCEVs@SPD (1 × 10¹¹ particles/mL) for 24 h. RNA sequencing was performed using three biological replicates per group (n = 3). Total RNA was extracted using TRIzol reagent and subjected to library construction and sequencing. Raw reads were quality filtered to remove adapters and low-quality sequences, followed by alignment to the reference genome. Gene expression levels were quantified and differential expression analysis was conducted using DESeq2. Differentially expressed genes were identified using: |log₂ fold change| ≥ 1; Benjamini–Hochberg FDR < 0.05. The RNA-seq dataset has been deposited in the NCBI Sequence Read Archive under accession number PRJNA1483132.

**Untargeted metabolomics analysis**

**Sample preparation and LC-MS acquisition**

RAW264.7 macrophages were stimulated with LPS (100 ng/mL) and IFN-γ (20 ng/mL) for 12 h, followed by treatment with RCEVs@SPD (1 × 10¹¹ particles/mL) for 24 h. Two groups were included: LPS control and RCEVs@SPD (n = 6 biological replicates per group). Cells were harvested, snap-frozen in liquid nitrogen, and stored at −80 °C prior to analysis. Metabolites were extracted using pre-chilled methanol/acetonitrile, and pooled quality control (QC) samples were prepared by mixing equal aliquots of each biological sample. QC samples were injected at regular intervals throughout the acquisition sequence to monitor analytical stability. Metabolomic profiling was performed using ultra-high performance liquid chromatography coupled with high-resolution mass spectrometry (UPLC–HRMS) operating in both positive and negative electrospray ionization modes. Data were acquired in full scan combined with data-dependent MS/MS acquisition.

**Data processing and quality control**

Raw LC-MS data were converted to mzML format using MSConvert and processed using XCMS (centWave algorithm) for peak detection, retention time correction, and alignment. Feature annotation was performed using MetaboAnnotation by matching accurate MS¹ m/z values (±10 ppm) to HMDB and KEGG databases. MS²-based identification was performed by matching fragmentation spectra against an in-house spectral library with a similarity score cutoff of 70%.

Quality control filtering included: Removal of features detected in <50% of QC samples or <80% of biological samples; K-nearest neighbor (KNN) imputation of missing values; Probabilistic quotient normalization (PQN); Removal of features with coefficient of variation (CV) >30% in QC samples. After filtering, high-quality features were retained for downstream analysis (positive mode: 11,507; negative mode: 7,811). PCA was performed to assess sample clustering and QC reproducibility.

**Differential metabolite analysis**

Normalized data were analyzed using Student’s t-test. P-values were adjusted for multiple comparisons using the Benjamini–Hochberg false discovery rate (FDR) method. Differential metabolites were defined as those meeting the following criteria:

Fold change ≥1.5 or ≤0.67; FDR-adjusted q-value <0.05; Variable importance in projection (VIP) >1.0 from supervised OPLS-DA. Biological interpretation and pathway enrichment analyses prioritized metabolites confirmed by MS² identification.

**Multivariate analysis and model validation**

Supervised orthogonal partial least squares discriminant analysis (OPLS-DA) was performed to assess group separation. Model robustness was evaluated using: R² (goodness of fit); Q² (predictive ability); 200-permutation testing. The model yielded R² = 0.899 and Q² = 0.645. Permutation testing demonstrated lower Q² values for permuted models and a negative Q² intercept, indicating absence of model overfitting. CV-ANOVA was performed to further validate model significance.

**Glucose Assay**

Intracellular glucose levels were measured using a Glucose Assay Kit (Beyotime, S0201S) according to the manufacturer’s instructions. Briefly, BMDM cells were seeded in 6-well plates and treated with RCEVs@SPD for 24 h. Cells were then washed twice with PBS and lysed using lysis buffer. After centrifugation at 12,000 × g for 5 min at 4 °C, the supernatants were collected. Samples or glucose standards were incubated with the assay reagent, and absorbance was measured at 630 nm using a microplate reader (Synergy H1, BioTek, USA). Intracellular glucose concentrations were calculated based on a standard curve.

**ATP Assay**

Intracellular ATP levels were measured using a chemiluminescence-based ATP Assay Kit (Beyotime, S0026) according to the manufacturer’s instructions. Briefly, BMDM cells were seeded in 6-well plates and treated with RCEVs@SPD for 24 h. Cells were washed twice with cold PBS and lysed with ATP lysis buffer. After centrifugation at 12,000 × g for 5 min at 4 °C, the supernatants were collected and mixed with ATP working solution in white opaque 96-well plates. Chemiluminescence was immediately measured using a multimode microplate reader. ATP concentrations were calculated based on a standard curve.

**L-Lactic Acid Assay**

L-lactic acid (LA) levels in culture supernatants and BMDM cells were measured using an L-Lactic Acid Colorimetric Assay Kit (Elabscience, E-BC-K044-M) according to the manufacturer’s instructions. BMDM cells were treated with RCEVs@SPD for 24 h. Culture supernatants were collected and centrifuged to remove debris. For intracellular LA measurement, cells were washed with cold PBS, lysed by ultrasonication on ice, and clarified by centrifugation. Protein concentrations were determined by BCA assay for normalization. Samples or standards were incubated with the enzyme working solution and chromogenic reagent, and absorbance was measured at 530 nm using a microplate reader. LA concentrations were calculated based on a standard curve.

**Extracellular acidification rate and Oxygen consumption rate measurement**

Extracellular acidification rate (ECAR) and oxygen consumption rate (OCR) were measured in BMDM cells using fluorometric assay kits. BMDMs were seeded into black clear‑bottom 96‑well plates and cultured overnight at 37 °C with 5% CO₂. Before measurement, the culture medium was gently removed and replaced with 100 µL saline; blank wells contained saline without cells. Cells were stimulated and incubated at 37 °C for 30 min protected from light. For ECAR measurement, 100 µL of ECAR working solution was added to each well, and fluorescence was recorded kinetically at 37 °C using a microplate reader (Ex/Em 490/535 nm) at 2-3 min intervals for 100-120 min. ECAR was calculated from the slope of the linear portion of the fluorescence-time curve (MCE, E‑BC‑F069).

For OCR measurement, 100 µL of OCR working solution was added to each well, and fluorescence was monitored kinetically at 37 °C using the excitation/emission settings recommended by the manufacturer. OCR was determined from the linear rate of fluorescence change over time and used as an indicator of mitochondrial oxygen consumption (MCE, E‑BC‑F062).

**Immunofluorescence staining**

Immunofluorescence staining was performed according to standard protocols. Briefly, adherent cells cultured on sterile glass coverslips or tissue sections were fixed with 4% paraformaldehyde (PFA) at room temperature for 15 min and washed three times with PBS. Samples were then blocked with 3% bovine serum albumin (BSA) for 30min at room temperature. Primary antibodies (listed in Table S2) diluted in blocking buffer were applied to the samples and incubated overnight at 4°C in a humidified chamber. On the following day, samples were washed three times with PBST and incubated with fluorophore-conjugated secondary antibodies for 1-2 h at 4°C in the dark. After washing, nuclei were counterstained with DAPI for 10 min at room temperature in the dark. Samples were mounted using an antifade mounting medium, and images were acquired using an upright fluorescence microscope.

**Western blot analysis**

BMDMs were lysed in RIPA buffer supplemented with protease and phosphatase inhibitor cocktails (CWBIO) on ice. Lysates were centrifuged at 12,000 × g for 15 min at 4 °C, and protein concentrations were determined using a BCA protein assay. Equal amounts of protein were separated by SDS-PAGE together with a prestained protein marker (Servicebio) and transferred onto 0.45 μm nitrocellulose (NC) membranes (Millipore). Membranes were blocked with 5% non-fat milk or bovine serum albumin (BSA) in Tris-buffered saline containing 0.1% Tween-20 (TBST) for 1 h at room temperature and then incubated with primary antibodies (listed in Table S2) overnight at 4 °C. After washing with TBST, membranes were incubated with horseradish peroxidase (HRP)-conjugated secondary antibodies for 1 h at room temperature. Protein bands were visualized using enhanced chemiluminescence (ECL) reagents and detected with a chemiluminescence imaging system. Band intensities were quantified using ImageJ software and normalized to the corresponding loading controls. Full-length, uncropped Western blot images corresponding to the main figures are provided in Supplementary Figures S12-S13.

**Construction of synovial organoids**

Synovial tissue samples were collected from two patients with RA undergoing joint replacement surgery, following approval by the Human Research Ethics Committee of Peking University Shenzhen Hospital. The harvested tissues were carefully dissected into approximately 1-2 mm³ fragments and subsequently embedded into pre-perforated bacterial cellulose scaffolds. The constructs were cultured in DMEM/F‑12 medium supplemented with 10% fetal bovine serum (FBS) and maintained at 37 °C in a humidified incubator containing 5% CO₂. The culture medium was refreshed every 72 hours.

**Construction of collagen-induced arthritis (CIA) model**

All animal procedures were conducted in strict accordance with the guidelines approved by the Animal Ethics Committee of Peking University Shenzhen Hospital (Approval No. 2023-566). Thirty-two male DBA/1J mice (8-10 weeks old) were randomly assigned to four groups (n = 8 per group): a non-immunized control group (Control), a disease model group (CIA), a treatment group receiving RCEVs@SPD, and a methotrexate-treated group (MTX; methotrexate purchased from Yuan Ye Biotech Co., Ltd., Shanghai, China). Randomization was performed using a computer-generated random number sequence after acclimatization. To induce CIA, bovine type II collagen (Cat. No. 20022, Chondrex, USA) was emulsified thoroughly with complete Freund’s adjuvant (CFA; Cat. No. 7002, Chondrex, USA) to form a stable emulsion. During primary immunization (Day 0), mice received a subcutaneous injection of the emulsion at the base of the tail. On Day 21, a booster immunization was administered subcutaneously using bovine type II collagen emulsified with incomplete Freund’s adjuvant (IFA) [3]. Arthritis development was monitored and scored every three days after the booster injection. Clinical severity was assessed using a standardized scoring system based on joint redness and swelling. Each paw was scored on a scale from 0 to 4 as follows: 0: No visible redness or swelling; 1: Mild swelling involving 1-2 digits, or slight swelling of a larger joint (ankle or wrist); 2: Swelling of more than two digits, or fulfillment of any two criteria listed under score 1; 3: Fulfillment of three criteria listed under score 1, or swelling of more than two digits accompanied by ankle or wrist swelling; 4: Swelling of more than two digits with pronounced swelling of the paw, ankle, or wrist. The maximum score per paw was 4, resulting in a maximum total score of 16 per mouse. Clinical scoring was performed independently by two investigators blinded to group allocation to minimize observer bias. The extent of disease progression was evaluated as described above and analyzed statistically among groups.

***In vivo* administration of RCEVs@SPD in CIA mice**

CIA was established in male DBA/1 mice as described above. Therapeutic intervention was initiated upon the onset of arthritis. Mice were randomly assigned to different treatment groups. RCEVs were isolated using sucrose cushion ultracentrifugation to improve vesicle purity. Endotoxin levels in the final RCEV preparations were quantified using a limulus amebocyte lysate (LAL) assay, yielding 0.173 ± 0.014 EU/mL (mean ± SD, n = 6), indicating minimal endotoxin contamination. All vesicle preparations were handled under sterile, endotoxin-free conditions using sterile reagents and buffers and were passed through 0.22 μm filters where applicable prior to in vivo administration.

RCEVs@SPD were administered via intra‑articular injection into the knee joint at a volume of 10 μL per joint, twice weekly. Each injection contained 1 × 10¹⁰ particles, as determined by nanoparticle tracking analysis (NTA). Based on the measured drug loading content (2.79%), the corresponding SPD dose per injection was calculated accordingly. RCEVs alone were administered at the same particle number (1 × 10¹⁰ particles per joint). Free SPD was intra‑articularly injected at an equivalent dose in the same volume (10 μL). Control animals received 10 μL sterile PBS. All intra-articular injections were performed using sterile syringes and needles under aseptic conditions. Clinical scores and paw thickness were monitored throughout the study. At the experimental endpoint, mice were sacrificed for micro‑CT, histological, and immunological analyses.

**Macrophage depletion *in vivo***

Macrophage depletion in DBA/1 mice was performed using Clodronate Liposomes (Yisheng Biotechnology, 40337ES05) following the manufacturer’s protocol. Mice received intravenous injections of 100 µL Clodronate Liposomes via the tail vein 6 h before CIA modeling and subsequently on days 0, 2, 4, and 6. Control animals were administered the same volume of PBS‑loaded liposomes. To evaluate depletion efficiency, flow cytometry was conducted on day 2, and histological and immunological analyses were performed on days 7 and 14. The efficacy of macrophage clearance was confirmed by quantifying CD11b⁺F4/80⁺ macrophages in spleen and synovial tissues using flow cytometry and immunofluorescence staining.

**Micro-CT analysis**

Bone specimens were fixed in 4% paraformaldehyde and subsequently subjected to micro-computed tomography scanning using a NEMO NMC-200 system (China). The acquired raw datasets were reconstructed with Recon 3D software to generate three-dimensional images. Regions of interest (ROI) were defined and analyzed with specialized quantitative software. Morphometric evaluation included the bone surface-to-tissue volume ratio (BS/TV). In addition, trabecular microarchitecture was assessed, and parameters such as trabecular thickness (Tb.Th) and trabecular number (Tb.N) were calculated based on the reconstructed 3D models.

**Statistical analysis**

All statistical evaluations were performed using GraphPad Prism version 10 (GraphPad Software Inc., San Diego, CA, USA). Quantitative measurements represent data acquired from at least three independent biological replicates, with representative results presented. Comparisons between two groups were conducted using unpaired Student’s t-test, while one-way ANOVA was applied for analyses involving more than two groups. Data are presented as mean values accompanied by standard deviation (mean ± SD). Two-tailed P values were used to assess statistical relevance, with significance thresholds defined as **p* < 0.05, ***p* < 0.01, ****p* < 0.001, *****p* < 0.0001, and 'ns' indicating non-significant differences.

Table S1. Primer sequences used for RT–qPCR analysis in the study.

| Real-time quantitative PCR primer sequence | |
| --- | --- |
| Gene | Sequence (5’- 3’ on minus strand) |
| *β-actin* | Fwd: CATTGCTGACAGGATGCAGAAGG |
|  | Rev: TGCTGGAAGGTGGACAGTGAGG |
| *iNOS* | Fwd: GAGACAGGGAAGTCTGAAGCAC |
|  | Rev: CCAGCAGTAGTTGCTCCTCTTC |
| *CD206* | Fwd: GTTCACCTGGAGTGATGGTTCTC |
|  | Rev: AGGACATGCCAGGGTCACCTTT |
| *GLUT1* | Fwd: GCTTCTCCAACTGGACCTCAAAC |
|  | Rev: ACGAGGAGCACCGTGAAGATGA |
| *HK2* | Fwd: CCCTGTGAAGATGTTGCCCACT |
|  | Rev: CCTTCGCTTGCCATTACGCACG |
| *ATP5A* | Fwd: TGGTGAAGAGACTGACGGATGC |
|  | Rev: TCAAAGCGTGCTTGCCGTTGTC |
| *ATP5B* | Fwd: CTCTGACTGGTTTGACCGTTGC |
|  | Rev: TGGTAGCCTACAGCAGAAGGGA |
| *LDHA* | Fwd: ACGCAGACAAGGAGCAGTGGAA |
|  | Rev: ATGCTCTCAGCCAAGTCTGCCA |
| *COX4I1* | Fwd: TCATTGGCTTCACTGCGCTCGT |
|  | Rev: TCCAGCATTCGCTTGGTCTGCA |
| *COX5B* | Fwd: TGCTACCTCCAAAGGCAGCTTC |
|  | Rev: CATCGCTGACTCTCGCCTTTGT |
| *NDUFA9* | Fwd: GTTTCACACCTGAATGCCAGTATG |
|  | Rev: GAACCTGTCCTCCCTTCCGAAA |
| *NDUFB8* | Fwd: CGCCAAGAAGTATAACATGCGAG |
|  | Rev: CCTCTCATGCTGTGATCGGTTG |
| *PGC-1α* | Fwd: GAATCAAGCCACTACAGACACCG |
|  | Rev: CATCCCTCTTGAGCCTTTCGTG |
| *PKM2* | Fwd: CAGAGAAGGTCTTCCTGGCTCA |
|  | Rev: GCCACATCACTGCCTTCAGCAC |
| *PFKFB3* | Fwd: TCATCGAGTCGGTCTGTGACGA |
|  | Rev: CATGGCTTCTGCTGAGTTGCAG |
|  |  |

Table S2. Antibodies used in the study.

| **Antibodies** | **Host Species** | **Source** | **Cat.** |
| --- | --- | --- | --- |
| ATP5A1 | Rabbit | Abclonal | A11945 |
| HK2 | Rabbit | Abclonal | A0994 |
| HIF-1α | Rabbit | Proteintech | 82989-4-RR |
| GLUT1 | Rabbit | Sellect | P22E15 |
| CD206 | Rabbit | Santa cruz biotechnology | SC-70585 |
| iNOS | Mouse | Santa cruz biotechnology | SC-7271 |
| PI3K | Rabbit | Affinity | AF6242 |
| p-PI3K | Rabbit | Affinity | AF3242 |
| AKT | Rabbit | CST | 4691 |
| p-AKT | Rabbit | CST | 4060 |
| ARG-1 | Rabbit | Affinity | DF6657 |
| CCR7 | Rabbit | Affinity | AF5293 |
| β-actin | Rabbit | Affinity | AF7018 |

Table S3. Physicochemical characteristics of plant extracellular vesicles isolated by ultracentrifugation and sucrose cushion methods.

| **Method** | **Average**  **particle size**  **(nm)** | **Concentration before membrane disruption（Particles/mL）** | **Concentration after membrane disruption（Particles/mL）** | **Purity (%)** |  |
| --- | --- | --- | --- | --- | --- |
| 1 | 83.4 | 6.84E+9 | 8.04E+8 | 88.2 |  |
| 2 | 75 | 2.1E+9 | 1.49E+8 | 92.9 |  |

Isolation methods: 1, ultracentrifugation; 2, sucrose cushion ultracentrifugation.

Table S4. Summary of LC–MS feature detection, MS² confirmation, and database annotation in positive and negative ion modes.

| **Mode** | **All** | **MS2** | **HMDB** | **KEGG** | **Annotated** |  |
| --- | --- | --- | --- | --- | --- | --- |
| Negative | 11891 | 580 | 7576 | 4741 | 7606 |  |
| Positive | 17180 | 538 | 13055 | 8009 | 13079 |  |

Table S5. Numbers of differential MS features between groups in positive and negative ion modes.

| **Comparision** | **Negative-down** | **Negative-up** | **Positive-down** | **Positive-up** |  |
| --- | --- | --- | --- | --- | --- |
| RCEVs@SPD/LPS | 339 | 413 | 514 | 503 |  |

Figure S1. Inflammatory activation drives glycolytic reprogramming and suppresses mitochondrial oxidative phosphorylation in RAW264.7 macrophages. Expression of OXPHOS-related genes and Glycolysis-associated genes.

Figure S2. Increased expression of inflammatory and glycolytic markers in synovial tissue of CIA mice. Representative immunohistochemical staining of synovial sections from healthy control mice and CIA mice showing the expression of iNOS (A) and GLUT1 (B). Quantification of the positively stained area is shown on the right for each marker.

Figure S3. Purity assessment of RCEVs. (A) Particle size distribution and concentration of RCEVs isolated by ultracentrifugation measured by NTA before and after membrane lysis. (B) Particle size distribution and concentration of RCEVs isolated by sucrose cushion centrifugation measured by NTA before and after membrane lysis.

Figure S4. UV-Vis characterization and standard calibration curve of spermidine (SPD). (A) UV-Vis absorption spectra of spermidine solutions at different concentrations (0.1-0.3 mg/mL). A characteristic absorption peak was observed at approximately 205 nm and used for quantitative analysis. (B) Standard calibration curve of spermidine constructed by plotting absorbance versus concentration. Linear regression analysis yielded the equation Y = 0.08400X + 1.104 with a correlation coefficient of R^2^ = 0.9983, demonstrating excellent linearity within the tested concentration range. This standard curve was used to quantify spermidine concentration for encapsulation efficiency (EE%) and loading content (LC%) calculations.

Figure S5. Cell viability of RAW264.7 macrophages treated with increasing concentrations of RCEVs at different time points was assessed using the CCK-8 assay. Cell viability is expressed as relative viability normalized to the control group.

Figure S6. HPLC-MS/MS analysis of intracellular SPD in cells following different treatments. (A) Representative chromatogram of endogenous SPD detected in untreated control cells, showing a characteristic peak at a retention time (RT) of approximately 4.22 min (m/z 146.088 → 84.126). (B) Chromatogram of cells treated with free SPD, exhibiting increased peak area compared with control cells. (C) Chromatogram of cells treated with RCEVs@SPD, showing a further increase in SPD peak intensity at a comparable retention time (RT≈4.21-4.22 min), indicating enhanced intracellular SPD accumulation. Quantitative data were calculated based on peak area integration under identical LC-MS/MS conditions.

Figure S7. Evaluation of cell viability after treatment with different endocytosis inhibitors. (A) Representative live/dead staining images of cells treated with various endocytosis inhibitors, including chlorpromazine, M-β-CD, filipin, and amiloride hydrochloride. Live cells are stained green and dead cells are stained red. The right column shows the merged images. Scale bar = 100 μm. (B) Quantitative analysis of cell viability based on live/dead staining. (C) Cell viability determined by CCK‑8 assay after treatment with different inhibitors. The results were normalized to the control group. Data are presented as mean ± SD (n = 6). ns indicates no statistically significant difference.

Figure S8. Representative fluorescence microscopy images showing the time-dependent intracellular distribution of CY5.5-labeled RCEVs (red) following incubation for 1, 2, 4, and 6 h. F-actin was stained with phalloidin (green) to visualize the cytoskeleton, and nuclei were counterstained with DAPI (blue). Merged images illustrate the progressive intracellular accumulation of RCEVs over time. Corresponding three-dimensional fluorescence intensity surface plots are shown on the right, providing a semi-quantitative visualization of the increasing intracellular CY5.5 signal intensity and spatial distribution. Scale bar, as indicated.

Figure S9. referential localization of RCEVs within macrophage populations in synovial organoids. (A, B) Representative confocal images of synovial organoids incubated with CY5.5‑labeled RCEVs (red) and stained for CD11b (green) and nuclei (DAPI, blue). Enlarged views of boxed regions are shown in the insets. Right panels: Line‑scan fluorescence intensity profiles along the indicated regions demonstrating highly coincident signal distributions between RCEVs and CD11b, supporting preferential macrophage localization. (C, D) Representative images of synovial organoids stained for FAP (green) following incubation with CY5.5‑labeled RCEVs (red). Insets show magnified views of selected regions. Right panels: Line‑scan analysis revealing comparatively reduced spatial coincidence between RCEVs and FAP signals relative to CD11b⁺ regions. Scale bars: 200 μm.

Figure S10. Multiex cytokine profiling reveals immunomodulatory effects of RCEVs and RCEVs@SPD. (A) Heatmap representation of cytokine expression profiles measured using a multiplex bead‑based cytokine detection kit and analyzed by flow cytometry. Data are normalized and presented as Z‑scores. (B) Quantitative analysis of IL‑6 levels expressed as mean fluorescence intensity (MFI). (C) Quantitative analysis of GM‑CSF levels (MFI). Data are presented as mean ± SD (n = 3). Statistical significance was determined by one‑way ANOVA with post hoc multiple comparisons. ***p* < 0.01.

Figure S11. RCEVs@SPD regulate macrophage metabolic reprogramming through modulation of the PI3K/AKT/AMPK signaling pathway. (A) Western blot analysis of PI3K, p‑PI3K, AKT, p‑AKT, AMPK, and p‑AMPK protein expression in macrophages treated with Control, RCEVs, SPD, or RCEVs@SPD. (B) Densitometric quantification of phosphorylation levels normalized to their corresponding total protein levels (p‑PI3K/PI3K, p‑AKT/AKT, and p‑AMPK/AMPK). (C) Western blot analysis of PI3K, p‑PI3K, AKT, and p‑AKT in macrophages treated with RCEVs@SPD in the presence or absence of the PI3K agonist 740Y‑P. (D) Quantitative analysis of p‑PI3K/PI3K and p‑AKT/AKT ratios showing that 740Y‑P restored PI3K and AKT phosphorylation levels, partially reversing the inhibitory effect of RCEVs@SPD on PI3K/AKT signaling. Data are presented as mean ± SD (n = 3 independent experiments). Statistical significance was determined by one‑way ANOVA with appropriate post hoc tests. **p* < 0.05, ***p* < 0.01, ****p* < 0.001, *****p* < 0.0001.

Figure S12. Uncropped raw Western blot images corresponding to Figure 5N and Figure S10A.

Figure S13. Uncropped raw Western blot images corresponding to Figure 4E and Figure S10C.

Figure S14. RCEVs@SPD enhance mitochondrial OXPHOS programs in macrophages. GSEA comparing RCEVs@SPD-treated versus control macrophages shows significant positive enrichment of oxidative-phosphorylation/ETC-related pathways, including Proton motive force-driven ATP synthesis, Aerobic respiration, Mitochondrial respiratory chain complex I, and Mitochondrial respirasome.

Figure S15. KEGG and GO enrichment of differentially expressed genes after RCEVs treatment. (A) KEGG over-representation analysis of up-regulated (left) and down-regulated (right) genes in RCEV-treated versus control macrophages. (B) GO enrichment for the same gene sets. Panels (top to bottom) correspond to Biological Process (BP), Cellular Component (CC), and Molecular Function (MF).

Figure S16. LC-MS data quality and feature coverage. (A-B) Total ion chromatograms (TICs) in negative (A) and positive (B) ESI modes across three biological replicates per group. Stable retention times and highly similar base-peak patterns indicate robust chromatography and acquisition reproducibility. (C-D) Two-dimensional feature maps (average retention time vs. m/z) for negative (C) and positive (D) modes with intensity heat scale and marginal distributions, showing broad coverage from early to mid/late RT windows and dense feature clusters within m/z 100–1,000.

Figure S17. MS¹-level identification depth and chemical class composition. (A) Distribution of the number of HMDB candidate matches per MS¹ feature in negative/positive modes, indicating extensive database coverage. (B) HMDB super-class composition for annotated features (negative/positive), dominated by lipids & lipid-like molecules, organic acids and derivatives, and organoheterocyclic compounds. (C) KEGG Level-2 functional categorization of all MS¹-matched features, with predominant assignments to amino-acid metabolism, lipid metabolism, and carbohydrate metabolism. (D) Distribution of the number of KEGG candidate matches per MS¹ feature (negative/positive).

Figure S18. MS²-level confidence, class-RT/m/z distribution, and pathway evidence. (A) RT-binned counts of super-classes highlighting temporal separation of lipids, organic acids/derivatives, and organoheterocyclic compounds. (B) RT-m/z scatter plots colored by chemical super-class (negative/positive), showing coherent separation with lipid species enriched at later RT. (C) Identification score distributions for MS²-confirmed features (negative/positive), skewed toward higher-confidence bins. (D–E) KEGG Level-2 classification and pathway assignment for MS²-identified differential metabolites, again concentrating in amino-acid, lipid, and carbohydrate metabolism. (F) KEGG enrichment bubble plot ranking central routes closely aligned with the main figures, including central carbon metabolism, oxidative phosphorylation, pantothenate and CoA biosynthesis, sulfur metabolism, aminoacyl-tRNA biosynthesis, alanine/aspartate/glutamate, arginine/proline, glycine/serine/threonine, purine metabolism, and ABC transporters.

Figure S19. KEGG functional classification and pathway enrichment analysis of differential metabolites. (A) Top enriched KEGG pathways ranked by the number of associated differential metabolites. (B) Distribution of differential metabolites across KEGG Level 2 metabolic pathways. (C) KEGG pathway enrichment analysis of differential metabolites, presented as a bubble plot based on rich factor, metabolite count, and adjusted *p* value. (D) KEGG functional classification of differential metabolites at Level 1 and Level 2, showing the distribution of metabolites across major biological categories.

Figure S20. MS¹/MS²-resolved metabolite alterations and pathway enrichment associated with metabolic reprogramming. (A) KEGG functional classification of MS¹-annotated differential metabolites at Level 1 and Level 2. (B) KEGG pathway enrichment analysis based on MS²-identified metabolites, highlighting signaling- and metabolism-related pathways.

Figure S21. Gross paw appearance and peripheral blood analysis in CIA mice after treatment.

Figure S22. Quantitative analysis of synovial inflammation and metabolic marker expression in CIA mice. (A) Quantification of intra-articular inflammatory cell area in ankle joint sections from Control, CIA, MTX, and RCEVs@SPD-treated mice. (B) Quantitative analysis of GLUT1-positive area (%) in ankle joint sections, reflecting synovial glycolytic activity. (C) Quantification of iNOS-positive area (%) in ankle joint sections, indicating pro-inflammatory macrophage activation.

Figure S23. Detection of pro-inflammatory cytokine levels in joint immune cells by flow cytometry. Quantitative MFI values of IL-1α, IL-1β, IL-6 and TNF-α across four groups. Data show mean ± SEM overlaid with individual replicates. *****p* < 0.0001, ns = not significant.

Figure S24. Biosafety evaluation of RCEVs@SPD. H&E staining of heart, liver, spleen, lung and kidney of mice at Day 43 after the administration of RCEVs@SPD.

Figure S25. Serum biochemical analysis of liver and renal function in CIA mice after treatment with RCEVs@SPD. (A) Serum levels of hepatic function biomarkers ALT, AST, and γ-GT in Control, CIA, MTX, and RCEVs@SPD groups. (B) Serum levels of renal function indicators UA, CREA, and UREA across the four experimental groups. All data are presented as mean ± SEM with individual animal data points overlaid. ns, no statistically significant difference among groups.

**Reference**

[1] C. Gao, Y. Zhou, Z. Chen, H. Li, Y. Xiao, W. Hao, Y. Zhu, C.T. Vong, M.A. Farag, Y. Wang, S. Wang, Turmeric-derived nanovesicles as novel nanobiologics for targeted therapy of ulcerative colitis, Theranostics 12(12) (2022) 5596-5614.

[2] R. Shi, W. Tan, H. Jin, S.I. Chan, W. Li, S.S. Lei, G. Cui, Y. Wang, D.H. Yang, Z. Zhong, MicroRNA-Enriched Plant-Derived Exosomes Alleviate Colitis by Modulating Systemic Immunity, Metabolic Homeostasis, and Gut Microbiota, Adv Sci (Weinh) 12(42) (2025) e05921.

[3] X. Wang, J. He, Y. Hong, L. Jie, B. Wu, J. Shen, G. Zhou, Q. Wang, Spermidine-encapsulated chondroitin sulfate methacryloyl hydrogel delivery system for remodeling bone homeostasis in rheumatoid arthritis, Mater Today Bio 35 (2025) 102531.
